# Chemical profiling of DNA G-quadruplex-interacting proteins in live cells

**DOI:** 10.1038/s41557-021-00736-9

**Published:** 2021-06-28

**Authors:** Xiaoyun Zhang, Jochen Spiegel, Sergio Martínez Cuesta, Santosh Adhikari, Shankar Balasubramanian

**Affiliations:** 1 Department of Chemistry, University of Cambridge, Cambridge, UK; 2 Cancer Research UK Cambridge Institute, Li Ka Shing Centre, University of Cambridge, Cambridge, UK; 3 School of Clinical Medicine, University of Cambridge, Cambridge, UK; 4 Present Address: Data Sciences and Quantitative Biology, Discovery Sciences, AstraZeneca, Cambridge, UK

**Keywords:** Chemical tools, Protein-protein interaction networks, Target identification, Chromatin

## Abstract

DNA–protein interactions regulate critical biological processes. Identifying proteins that bind to specific, functional genomic loci is essential to understand the underlying regulatory mechanisms on a molecular level. Here we describe a co-binding-mediated protein profiling (CMPP) strategy to investigate the interactome of DNA G-quadruplexes (G4s) in native chromatin. CMPP involves cell-permeable, functionalized G4-ligand probes that bind endogenous G4s and subsequently crosslink to co-binding G4-interacting proteins in situ. We first showed the robustness of CMPP by proximity labelling of a G4 binding protein in vitro. Employing this approach in live cells, we then identified hundreds of putative G4-interacting proteins from various functional classes. Next, we confirmed a high G4-binding affinity and selectivity for several newly discovered G4 interactors in vitro, and we validated direct G4 interactions for a functionally important candidate in cellular chromatin using an independent approach. Our studies provide a chemical strategy to map protein interactions of specific nucleic acid features in living cells.

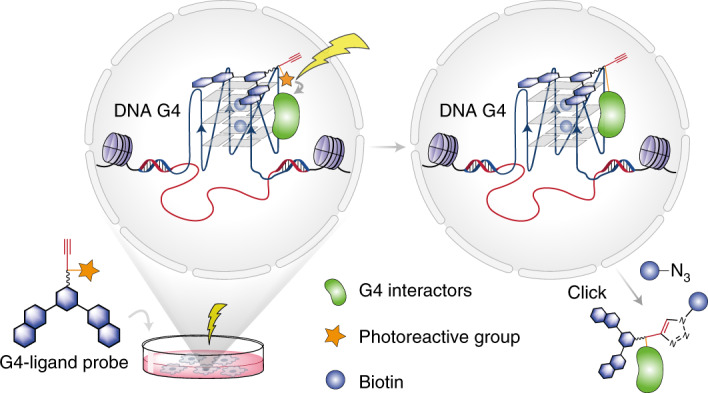

## Abstract (web summary)

DNA–protein interactions are essential to genome function, but they are challenging to map in a cellular environment. Now, a chemical proteomics approach, which uses DNA G-quadruplex-specific ligands containing a photocrosslinking motif, has enabled the systematic identification of DNA G-quadruplex-binding proteins in live cells.

## Main

Intricate networks of direct and coordinated interactions between proteins and nucleic acids are of vital importance in the regulation of numerous cellular processes, such as gene expression, DNA replication or DNA repair^1^. Robust methods that can interrogate these interaction networks in a native chromatin context are key to understand the underlying molecular mechanisms^2,3^. Chromatin immunoprecipitation (ChIP) has been coupled with mass spectrometry (MS)-based proteomics analysis to characterize the composition of particular chromatin-associated protein complexes^4–6^. However, these approaches require high-affinity and high-selectivity antibodies that typically explore one protein of interest at a time. Alternatively, enzyme-catalysed proximity labelling approaches, such as BioID or APEX, target promiscuous labelling enzymes to specific proteins of a subcellular compartment by genetic fusion, by which they promote the covalent tagging of endogenous neighbouring proteins^3,7^. Despite several successful examples, applicability and spatial resolution can be hindered by relatively slow labelling kinetics, toxicity and the size of the engineered fusion proteins^8^.

In contrast, photoactivation of small-molecule crosslinkers allows for a precise control of the reaction and shorter labelling times to provide relatively low background binding and good spatial and temporal resolution^9^. In affinity-based protein profiling, small molecules are linked to photocrosslinkers that mediate the irreversible binding to cellular protein targets in situ, followed by characterization via quantitative proteomics^10,11^. However, such approaches have so far been used to map direct protein interactors of drugs or small-molecule fragments^12,13^ rather than interaction networks. Thus, novel strategies that circumvent these limitations and provide a more holistic view of protein interactions at particular functional genomic sites are highly required.

**Fig. 1:**
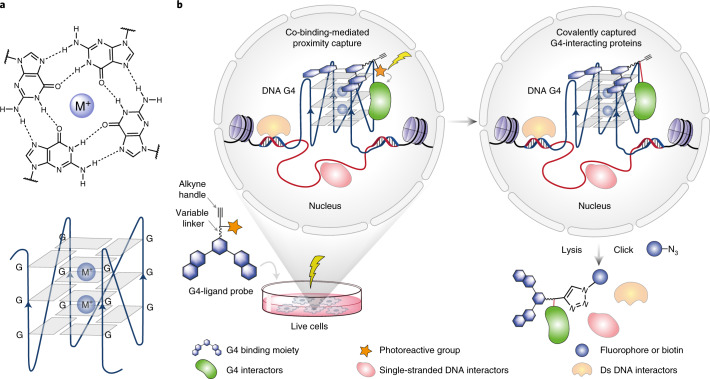
Schematic for CMPP. **a**, A G-tetrad stabilized by Hoogsteen base pairing and a monovalent cation (top), and an intramolecular G4 structure formed by the stacking of G-tetrads (bottom). **b**, Schematic representation of the CMPP concept. Cells are treated with G4-ligand probes that are functionalized with a photoreactive diazirine group and a click alkyne handle. The probes are recruited to endogenous G4 binding sites, where ultraviolet irradiation triggers the proximity capture of co-binding G4-interacting proteins.

DNA G-quadruplexes (G4s) are non-canonical, four-stranded nucleic acid structures that comprise stacked G-tetrads within certain G-rich sequences (Fig. 1a)^14,15^. DNA G4s have been shown to exist in human cells^16–18^, and their formation is dynamic in live cells^19^. G4 sequencing (G4-seq) identified more than 700,000 sites in human genomic DNA that have the biophysical potential to form G4s (potential G4s)^20^. G4 chromatin immunoprecipitation sequencing (G4 ChIP-seq)^21^ found endogenous DNA G4s enriched in open chromatin regions and promoters of highly expressed cancer genes^22^, and these G4s were recently linked to underlying transcription factor programmes in breast cancer^23^. Notably, the formation of endogenous G4s is cell-type specific with only 1% (~10,000 sites) of the in vitro potential G4s^20^ being detected in chromatin^21^. Taken together, these data suggest that G4 folding in chromatin is dynamic and that G4 homeostasis and functions may be intricately linked to interacting proteins^24^. A variety of proteins, such as helicases^25,26^, transcription factors^27–29^ and epigenetic modulators^30^, have been shown to interact with DNA G4s in vitro. However, DNA G4 binding proteins have mostly been explored by affinity enrichment from lysed samples using synthetic G4 oligonucleotides as baits^31–33^. Such affinity purification experiments do not account for the native chromatin environment, which is intricately linked to G4 biology^22^.

Here, we report a co-binding-mediated protein profiling (CMPP) approach for the investigation of DNA G4-interacting proteins in living cells. In this strategy, functionalized small-molecule ligands are designed to bind G4 structures in cellular chromatin, which serve as docking sites to bring the probes into close proximity to the G4-interacting proteins and enable labelling by subsequent photocrosslinking (Fig. 1b). We first showed that this concept can be efficiently applied with minimal perturbation of G4-protein interactions by photoproximity crosslinking of a G4-binding antibody in vitro. We then employed this approach in human cells to identify hundreds of putative G4-interacting proteins that comprised diverse functional classes. Next, we characterized the G4 binding properties for a representative set of proteins in vitro and found strong and selective G4 binding interactions for several of the novel candidates. Lastly, we further investigated one of the candidates, the chromatin remodeller SMARCA4, and revealed its recruitment to endogenous promoter G4s in chromatin.

## Results

### Design of co-binding-mediated protein profiling

A small molecule that binds a variety of G4 DNA target structures in cells could be functionalized to allow mapping of G4-interacting proteins in their native environment with minimal perturbation (Fig. 1b). We based our probe design on pyridostatin (PDS), a highly G4-selective small-molecule ligand that has been widely used to target DNA and RNA G4s in cells^34^. We previously showed that a PDS derivative and a protein can simultaneously bind a G4 in vitro^35^, which makes a promising molecular scaffold to detect co-binding proteins.

**Fig. 2:**
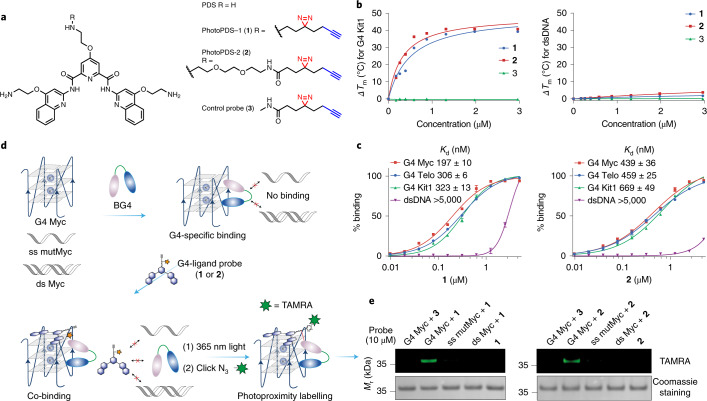
Co-binding-mediated proximity capture of a G4 binding protein in vitro. **a**, Chemical structures of G4-ligand probes photoPDS-1 (1), photoPDS-2 (2) and the control probe 3. **b**, Thermal melting shifts of G4 Kit1 (left) and dsDNA (right) induced by increasing concentrations of 1, 2 and 3. The increase in melting temperature (Δ*T*_m_) was measured by a fluorescence resonance energy transfer melting assay. The mean is from two independent experiments (*n* = 2). **c**, Fluorescence quenching induced by increasing the concentrations of probes 1, 2 and 3 bound to different G4 structures (G4 Myc, G4 Kit1 and G4 Telo) and dsDNA. The apparent *K*_d_ values are shown. Mean and error (± standard deviation (s.d.)) are from four independent experiments (*n* = 4). **d**, Schematic representation of the co-binding-mediated proximity capture of BG4 in vitro. **e**, Gel scans (probe, 10 μM) showing fluorescence images of co-binding-mediated proximity labelling of BG4 (molecular mass ~31 kDa) by 1, 2 and 3. Representative images from three independent experiments with similar results are shown. Source data

We prepared two G4-ligand probes, photoPDS-1 (1) and photoPDS-2 (2) (Fig. 2a), by tethering PDS to a click alkyne handle and a photoreactive aliphatic diazirine group, which is small and has excellent chemical stability, photolabelling efficiency and low background binding^36,37^. Probe 1 has a short, two-carbon linker and probe 2 has a two-unit polyethylene glycol longer linker (12 atoms) to enable probing proteins at different distances from the G4 binding site. In addition, we prepared a photoactivatable control 3 (Fig. 2a) that lacks a G4 binding moiety.

**Extended Data Fig. 1:**
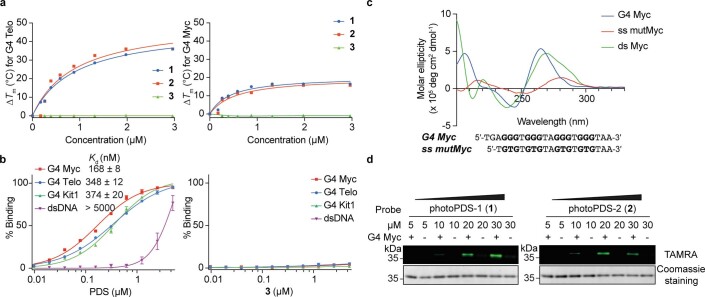
Probes for co-binding-mediated proximity labelling of BG4 in vitro. **a**, Assessment of G4-ligand probes (1-3) of inducing thermal stabilization (Δ*T*_m_) on G4 Telo and G4 Myc using FRET melting assay. Δ*T*_m_ of 1 and 2 at 1 μM on G4 Telo are 25 °C and 27 °C, respectively. Δ*T*_m_ of 1 and 2 at 1 μM on G4 Myc, are 14 °C and 13 °C, respectively. While Δ*T*_m_ of 3 at 1 μM is 0. Mean is represented from two independent experiments (n = 2). **b**, Assessment of G4-binding affinity of PDS and 3 using fluorescence titration binding assay by measuring apparent *K*_d_ values. Mean and error (± S.D.) are represented from four independent experiments (n = 4). **c**, Structure verification of G4 Myc, single-stranded mutMyc and double-stranded Myc with circular dichroism (G-runs are highlighted in bold). Mean of three independent experiments (n = 3) is represented. **d**, Dose-dependent of CMPP of BG4 by 1 and 2. Signals from TAMRA and Coomassie staining represent probe-specific labelling and loading input, respectively. Representative images from three independent experiments with similar results are shown. Source data

First, we assessed the binding affinity and selectivity of the probes towards G4 structures using an established fluorescence resonance energy transfer melting assay^38^. Compared with the parent compound PDS, both 1 and 2 retained the capacity to bind and stabilize a panel of G4 oligonucleotides (G4 Kit1, G4 Myc and G4 Telo) (Supplementary Table 1) and showed negligible stabilization of double-stranded DNA (dsDNA) (Fig. 2b and Extended Data Fig. 1a). Furthermore, fluorescence quench binding assays^39^ confirmed that 1 and 2 exhibit strong and selective binding to different G4 structures (Supplementary Table 2), such as G4 Myc with an apparent dissociation constants (*K*_d_) of 197 ± 10 nM and 439 ± 36 nM, respectively (Fig. 2c), comparable to that of PDS binding (*K*_d_ = 168 ± 8 nM; Extended Data Fig. 1b). In contrast, 3 showed no apparent G4 binding (Fig. 2b and Extended Data Fig. 1a,b).

### Photoproximity labelling of a G4 binding protein in vitro

As a proof of concept, we tested the probes using the G4-specific antibody BG4^17^ in vitro (Fig. 2d). BG4 was incubated with a folded G4 Myc oligonucleotide that forms a well-characterized G4 structure, as well as incubation with non-G4 control oligonucleotides, such as a mutated single-stranded Myc (ss mutMyc) and a double-stranded Myc (ds Myc). The presence or absence of G4 formation was confirmed by circular dichroism spectroscopy (Extended Data Fig. 1c). Probes 1 and 2, as well as control 3, were then incubated with the pre-incubated BG4–oligonucleotides mixtures and photocrosslinked at 365 nm. For each case, the probe was subsequently conjugated with tetramethylrhodamine-azide (TAMRA-azide) via the copper-catalysed azide–alkyne cycloaddition click reaction^40^, and the protein–oligonucleotide–probe mixtures were each separated by denaturing sodium dodecyl sulfate–polyacrylamide gel electrophoresis (SDS–PAGE) and then visualized by in-gel fluorescence scanning. We observed dose-dependent labelling of G4-Myc-bound BG4 by both probes 1 and 2 (Fig. 2e and Extended Data Fig. 1d), whereas negligible labelling was observed for control 3 (Fig. 2e). In addition, no labelling was observed in the presence of the control oligonucleotides ss mutMyc and ds Myc or in the absence of an oligonucleotide. This demonstrates for both probes 1 and 2 that crosslinking is made possible by co-binding to a G4 structure. In the case of BG4, labelling by probe 1 with the short linker, also suggests that the probe and BG4 co-bind to G4s in close proximity. The proof-of-concept paved the way for experiments to identify G4 binding proteins in cells.

### Global profiling of DNA G4-interacting proteins in cells

**Fig. 3:**
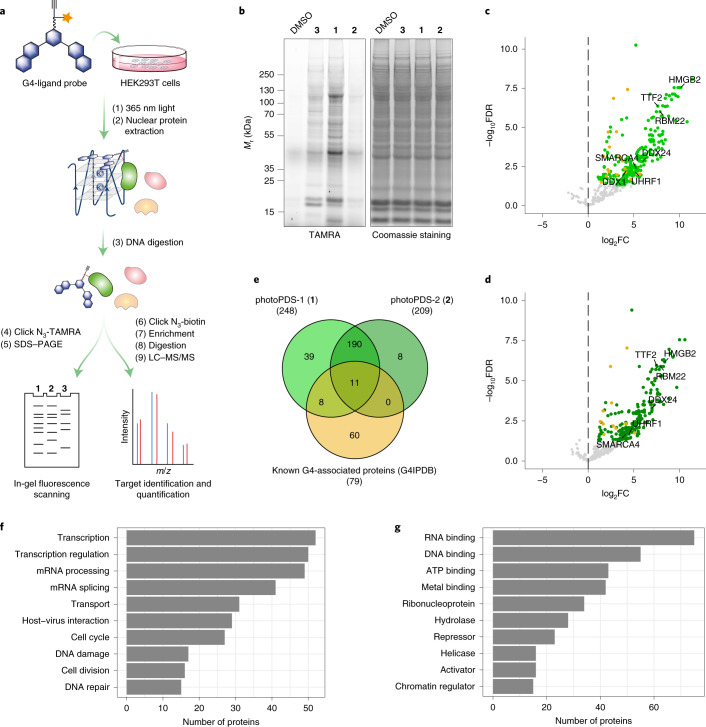
Profiling of G4 interactomes in human cells. **a**, Schematic workflow of the in situ mapping of G4-interacting proteins in HEK293T cells. **b**, Gel-based global profiling of G4-interacting proteins using probes (20 μM) 1 and 2 versus 3. TAMRA and Coomassie staining represent probe-specific labelling and total loading proteins, respectively. A representative image from three independent experiments with similar results is shown. **c**, Volcano plot displaying enriched proteins (highlighted in green and orange, respectively) for probe 1 versus 3 (*n* = 248). **d**, Volcano plot displaying enriched proteins (highlighted in green and orange, respectively) for probe 2 versus 3 (*n* = 209). Proteins were considered enriched with a >2-fold signal over control and a FDR <0.05. **e**, Overlap between enriched proteins in **c** and **d** in comparison with the known G4-associated proteins as available in G4IPDB^41^. Orange dots in **c** and **d** represent the enriched known G4-associated proteins. **f**,**g**, Distribution of the top UniprotKB keywords for biological process (**f**) and molecular function (**g**) of all the enriched proteins (256). DMSO, dimethylsulfoxide. Source data

**Extended Data Fig. 2:**
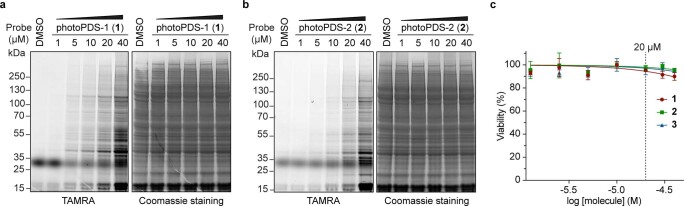
Gel-based mapping of DNA G4-interacting proteins in human cells. **a**, Probe 1 and **b**, probe 2 display dose-depend protein labelling of nuclear proteomes in HEK293T cells. Representative gel images from three independent experiments with similar results are shown. **c**, CellTiter-Glo luminescent cell viability assay on probe treatment for 75 min to HEK293T cells under all conditions used in this study. Mean and error (± S.D.) are represented from four independent experiments (n = 4). Source data

We next employed our approach to identify G4-interacting proteins in human cells. Embryonic kidney HEK293T cells were treated with probes 1 and 2, and control 3 (20 μM), followed by photocrosslinking at 365 nm. The nuclear extract was conjugated with TAMRA-azide via the copper-catalysed azide–alkyne cycloaddition reaction, separated by SDS–PAGE and visualized by in-gel fluorescence scanning (Fig. 3a)^13^. We observed distinct bands over a range of concentrations for both probes 1 and 2 (Fig. 3b and Extended Data Fig. 2a,b), which confirmed specific protein labelling as well as a good cell permeability and nuclear uptake, although probe 1 displayed a slightly higher efficiency. In addition, the probes did not show cell toxicity under the treatment conditions employed (Extended Data Fig. 2c).

Next, to identify the target proteins captured by G4-ligand probes, we employed a label-free, quantitative liquid chromatography (LC)–MS proteomics approach^4^. After photocrosslinking and extraction of the nuclear lysate, proteins were conjugated to biotin-azide and affinity purified on streptavidin beads, followed by on-bead digestion and quantitative LC–MS/MS analysis (Fig. 3a). Proteins that were detected in at least two out of four biological replicates and appeared significantly enriched over the non-specific probe 3 (fold change (FC) >2, false discovery rate (FDR) <0.05) were considered as candidate G4-interacting proteins. In total, we obtained 248 and 209 enriched protein targets for 1 and 2, respectively, from diverse functional classes (Fig. 3c,d). Interestingly, probe 2 shares ~96% (201 out of 209) of candidates with 1 (Fig. 3e), which suggests the linker length was not critical, in line with our observations for single protein BG4 labelling in vitro. Some of the candidate G4-interacting proteins overlapped with previously reported G4-interacting proteins^41^ for both probes 1 (19/79, 24%) and 2 (11/79, 14%), which provides independent corroboration for some of the findings, as well as new candidates, with our method.

Analysis of the annotated biological processes (Methods) revealed that the identified candidates are implicated in various different nuclear processes (Fig. 3f). In particular, we observed a large number of proteins involved in transcription, which is consistent with the emerging role of DNA G4s in transcriptional regulation^24^. Among the enriched proteins from diverse functional classes (Fig. 3g), we identified 19 of previously reported G4 interactors, such as hnRNP A1^42^ and nucleolin^32^. Importantly, we identified numerous novel candidate G4 interactors, such as a master epigenetic regulator UHRF1, transcription termination factor TTF2, ATP-dependent RNA helicases (for example, DDX1 and DDX24) and pre-mRNA-splicing factor RBM22, that have been shown to have a direct association with chromatin^43^. Interestingly, we also identified several subunits of the chromatin remodelling complex SWI/SNF (SWItch/sucrose non-fermentable), such as SMARCA4 and SMARCC1, which have only recently been linked to DNA G4s^31,44^.

### Characterization of candidate proteins in vitro

**Fig. 4:**
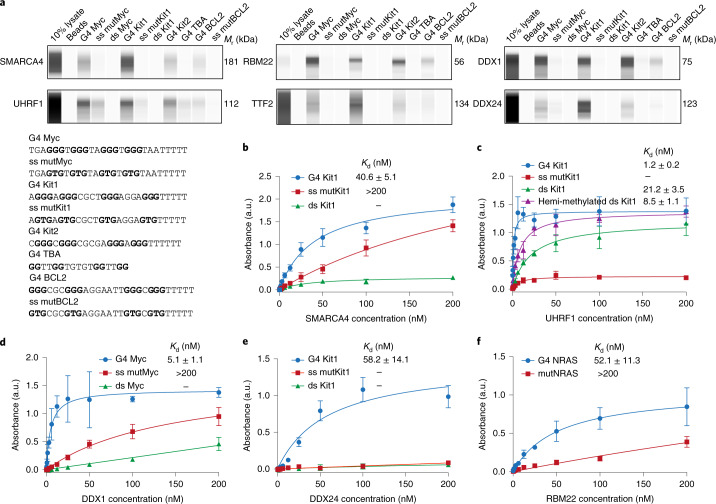
Validation of novel nuclear G4-selective binding proteins. **a**, Affinity enrichment coupled with western blot analysis of selected candidates for different topologies of G4 structures and control oligonucleotides (G-runs are highlighted in bold). A representative blot from two independent experiments with similar results is shown. **b**–**e**, Binding curves (the indicated *K*_d_ values were generated by ELISA) for the human recombinant full-length SMARCA4 protein to G4 Kit1, the single-stranded mutant (ss mutKit1) and double-stranded Kit1 (ds Kt1) (**b**), UHRF1 protein to G4 Kit1, ss mutKit1, Kit1 hemi-methylated dsDNA and ds Kit1 (**c**), DDX1 protein to G4 Myc, ss mutMyc and ds Myc (**d**), DDX24 protein to G4 Kit1, ss mutKit1 and ds Kit1 (**e**) and RBM22 protein to G4 NRAS and its mutant (mutNRAS) (**f**). Mean and error (± s.d.) are from three independent experiments (*n* = 3). a.u., arbitrary units. Source data

**Extended Data Fig. 3:**
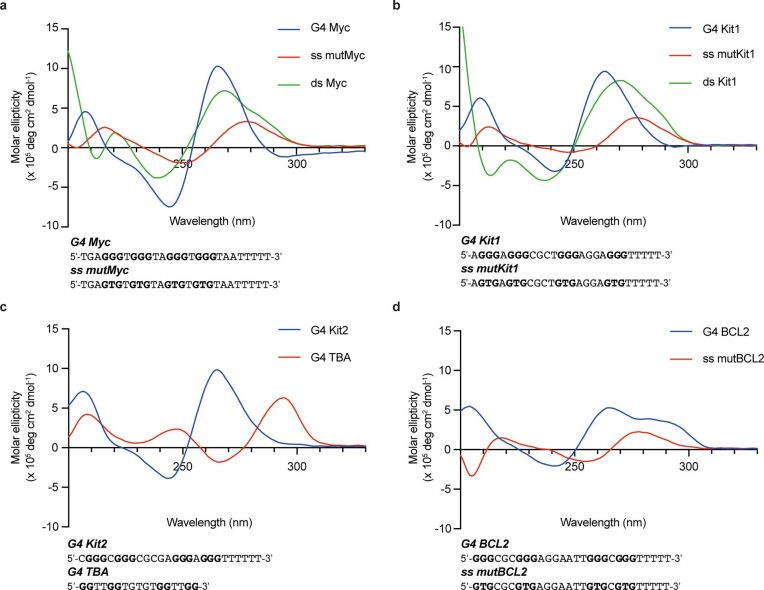
Structure verification of oligonucleotides. CD spectra obtained here match previously reported spectra of the well-characterized DNA G4 sequences (G-runs are highlighted in bold, see Supplementary Table 3) with different topologies showing distinct bands^65,66^, including parallel **a**,G4 Myc **b**, G4 Kit1 and **c**, G4 Kit2 by positive at ~260 nm and negative at ~240 nm; anti-parallel G4 TBA by positive at ~290 nm and ~240 nm, and negative at ~260 nm; **d**, hybrid G4 BCL2 by positive at ~290 nm and ~260 nm, and negative at ~240 nm. All G4 structures also share a positive band at ~210 nm. While the corresponding single-stranded mutant and duplex controls have lost these features. Mean of three independent experiments (n = 3) is represented. Source data

**Extended Data Fig. 4:**
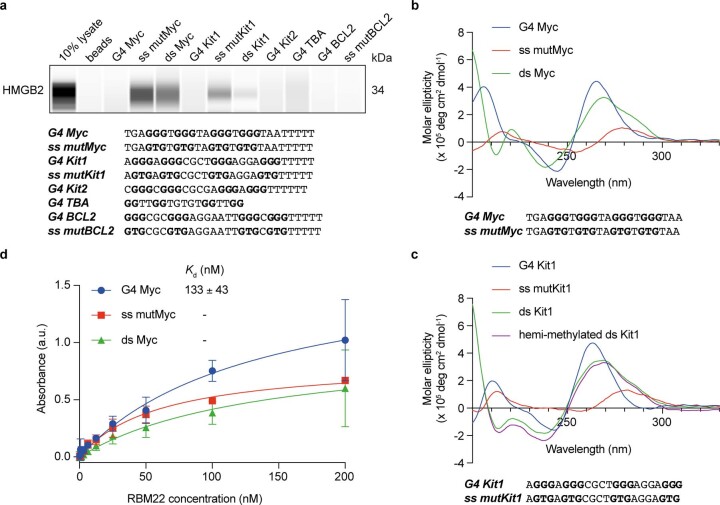
Protein validation by affinity enrichment coupled with western blot analysis and ELISA. **a**, Affinity enrichment coupled with western blot analysis of HMGB2 for different topologies of G4 structures and control oligonucleotides. A representative blot from two independent experiments with similar results is shown. Structure verification of G4 Myc (**b**) and G4 Kit1 (**c**) and the indicated control oligonucleotides with CD spectroscopy. Curves are plotted by mean values of three independent experiments (n = 3). **d**, Binding curves with indicated dissociation constants (*K*_d_) generated by ELISA for human recombinant full-length RBM22 protein to DNA G4 Myc, single-stranded mutant and Myc duplex DNA. Mean and error (± S.D.) are represented from three independent experiments (n = 3). G-runs are highlighted in bold. Source data

Candidate G4-interacting proteins identified by co-binding-mediated proximity labelling could potentially bind to G4 directly or as part of a protein complex bound to G4 or in close proximity to G4s. To better characterize the binding properties for a selection of candidate proteins, we employed a selection of 3′-biotinylated, well-characterized G4 oligonucleotides that can form different types of G4 structures, which include parallel (Myc, Kit1 and Kit2), antiparallel (TBA) and hybrid (BCL2) G4s (Supplementary Table 3). The corresponding mutated single-stranded mutant sequences that cannot fold into G4s and dsDNA were used as controls (Extended Data Fig. 3). The oligonucleotides were immobilized on streptavidin beads and used to affinity-enrich target proteins from HEK293T nuclear lysates, followed by western blot analysis. We investigated a selection of candidates identified by CMPP (SMARCA4, UHRF1, RBM22, TTF2, DDX24, DDX1 and HMGB2) that represent a variety of different functional protein classes (Fig. 3c,d). Strikingly, six out seven candidates showed G4-specific binding compared with that of the corresponding controls (Fig. 4a and Supplementary Table 4). One protein, HMGB2, displayed single-stranded DNA and dsDNA, but no G4 binding (Extended Data Fig. 4a–c), which indicates that HMGB2 may bind to the dsDNA adjacent to G4s or to the single-stranded opposite strand. Intriguingly, all the other six G4 binding proteins displayed selectivity for different G4 topologies. Although SMARCA4, TTF2 and DDX24 each showed a preference for a particular G4 sequence, RBM22, UHRF1 and DDX1 bound equally strongly to all parallel G4s (Myc, Kit1 and Kit2) and well to hybrid-type G4 (BCL2) (Fig. 4a). Importantly, our findings for DDX1 are in line with its reported G4 binding affinity, which validates the approach^45^. Notably, RBM22 showed a particularly high enrichment of relative intensity for G4s (Myc, Kit1, Kit2 and BCL2) compared with that of the 10% lysate control (Fig. 4a and Supplementary Table 5).

In principle, these affinity-enrichment experiments cannot distinguish direct G4 binders from proteins that are co-precipitated. Therefore, we carried out enzyme-linked immunosorbent assays (ELISAs) to assess the binding affinities for a selection of purified recombinant proteins (SMARCA4, UHRF1, DDX1, DDX24 and RBM22) (Supplementary Table 6). All five candidates displayed selective and high-affinity binding to G4s. SMARCA4 bound G4 Kit1 with *K*_d_ = 40.6 ± 5.1 nM (Fig. 4b). UHRF1 showed tight binding to G4 Kit1 with *K*_d_ = 1.2 ± 0.2 nM, which is more than 7-fold lower than that of its known substrate hemi-methylated dsDNA (*K*_d_ = 8.5 ± 1.1 nM) and 20-fold lower than its unmethylated duplex control (*K*_d_ = 21.2 ± 3.5 nM) (Fig. 4c). Similarly, DDX1 and DDX24 showed a low nanomolar affinity to G4 Myc (*K*_d_ = 5.1 ± 1.1 nM) and Kit1 (*K*_d_ = 58.2 ± 14.1 nM), respectively (Fig. 4d,e). RBM22 selectively bound to both DNA and RNA G4s and a preference for RNA NRAS G4 (*K*_d_ = 52.1 ± 11.3 nM) was observed (Fig. 4f and Extended Data Fig. 4d). Consistent with the affinity-enrichment experiments, considerably weaker or negligible binding was observed towards the control oligomers.

The affinity enrichment coupled with western blot analysis and ELISA experiments confirmed that our novel CMPP approach identifies genuine G4-interacting proteins in cells.

### SMARCA4 binds at endogenous G4 in chromatin

Chromatin architecture is tightly linked to the presence of endogenous DNA G4s^22^ and may affect the binding of protein interactors. To further validate G4 binding interactions in a chromatin context, we focused on the candidate interactor SMARCA4, which is a part of the SWI/SNF chromatin remodelling complex that plays a key role in transcriptional regulation^46^. Given that endogenous G4s have recently been mapped to open chromatin regions and promoters of highly expressed genes^22^, SMARCA4 may be linked to G4 function.

**Fig. 5:**
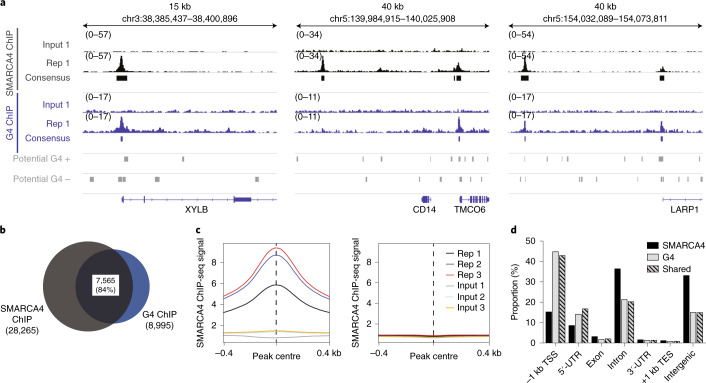
SMARCA4 is enriched at endogenous G4s. **a**, Example genome browser view for *XYLB*, *TMCC6* and *LARP1*. Signal tracks from ChIP-seq and control input as well as consensus peaks are shown for SMARCA4 (black) and G4s (blue). Sequences that have the biophysical potential to form G4s are shown for plus and minus strands (potential G4s, grey). **b**, Overlap of SMARCA4 and endogenous G4 high-confidence peaks. **c**, Occupancy profiles of SMARCA4 endogenous G4 sites (left) and potential G4s (right). **d**, Proportion of SMARCA4 and G4 ChIP-seq peaks across different genomic features. TSS, transcription start site; UTR, untranslated region; TES, transcription end site; Rep, replicate. Source data

**Extended Data Fig. 5:**
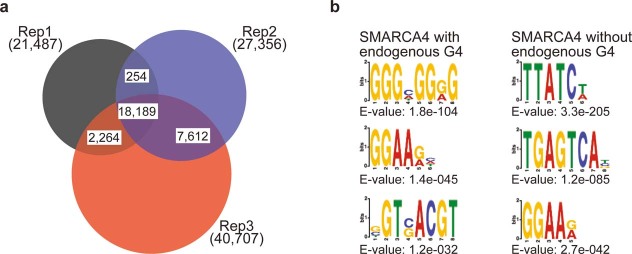
Properties of SMARCA4 binding sites. **a**, Overlap of binding sites identified by SMARCA4 ChIP-seq in K562 chromatin across three biological replicates. Binding sites identified in at least two replicates were considered as high confidence binding sites. **b**, Binding motifs identified in SMARCA4 binding sites that are marked by or lack and endogenous G4. The top3 motifs identified by EM for Motif Elicitation (MEME)^67^ analysis are shown.

We focused on human K562 chronic myelogenous leukaemia cells in which we previously mapped endogenous G4s via G4 ChIP-seq^21,30^. In this cell line, we performed SMARCA4 ChIP-seq and identified 28,265 SMARCA4 high-confidence binding sites from three biological replicates (Extended Data Fig. 5a). Strikingly, we observed that the majority of endogenous G4s (7,565 of 8,995, 84%) overlapped with SMARCA4 binding sites (Fig. 5a,b). Moreover, the SMARCA4 ChIP-seq signal was highly enriched and centred on endogenous G4 sites supportive of a direct SMARCA4-G4 binding interaction in chromatin (Fig. 5c). In contrast, no particular signal enrichment was observed at control sites that have the biophysical potential to form G4 single-stranded human DNA (potential G4s)^20,47^, but do not actually form folded G4 structures in chromatin for this cell line (Fig. 5c). Thus, the data show SMARCA4 binds to folded G4 secondary structures in chromatin, but not to the underlying G-rich dsDNA primary sequence in chromatin.

Investigating SMARCA4 binding sites at different functional genomic regions, we observed the largest proportion of SMARCA4-G4 co-localization at promoters (42% of peaks), which suggests that these interactions may play a particular role in SMARCA4 promoter activity (Fig. 5d)^48^. In addition, although most SMARCA4 binding sites contained A/T-rich motifs (Extended Data Fig. 5b), a dominant G-rich motif was found in binding sites marked by endogenous G4s, which supports a direct binding to G4 structures and indicates an important alternative mode of recruitment to chromatin.

## Discussion

Here we present a chemical CMPP approach to identify the cellular interactome of DNA G4 structures in native chromatin. The method employs functionalized, structure-specific small-molecule ligands that bind to G4s and mediate proximity labelling of endogenous G4 binding proteins via photoactivatable diazirine groups. Compared with proteomic approaches carried out in vitro, the in situ capture in cells takes into account the local chromatin environment in a functioning cell and should also facilitate the detection of transient G4-protein interactions that are lost during cell lysis or washing steps^7^.

Using the approach, we identified several hundred G4-associated proteins of which some were known G4-binders and many were not previously described. Several new G4 binding proteins were separately validated by in vitro assays and shown to be specific, high-affinity G4 binders. Given their distinct properties and various functions in biological processes, these proteins may play different key roles in regulation of the endogenous G4 landscape and G4 biology. The protein SMARCA4, which is part of a chromatin remodelling complex, was followed up further using genomic ChIP-seq methodology to demonstrate that SMARCA4 does, indeed, bind substantially to genomic sites in which G4 structures have been detected. This outcome confirms that our CMPP methodology does identify proteins that bind to G4 structures in cellular chromatin, particularly at gene promoters, and also implicates that SMARCA4-G4 interactions may be important for transcriptional control. Further experiments that involve protein knockdown or overexpression coupled with G4 ChIP-seq may ultimately help elucidate the associated mechanisms in more detail.

Although the CMPP probes were employed for relatively short treatment times, we cannot rule out the possibility that the ligands partially influence the endogenous G4 landscape and interactome. In this study and in other work^35^, PDS and G4-interacting proteins have been shown to co-bind to the same G4 structure; however, the situation can be more complex at high PDS concentrations, in which it has been shown to inhibit the binding of certain proteins to G4s^34,49^. In addition, G4 ligands may induce the stabilization of weaker, more transient G4s or alter the folded topology of G4s in ways that may influence protein binding. For these reasons it is essential to validate candidate G4 interactors with orthogonal approaches in vitro and in untreated cells, as we show in this study. We were mindful of observations that prolonged treatment with G4 ligands can induce DNA damage and recruit associated proteins^16^. Therefore, we limited ligand treatment times and concentrations to avoid potential artefacts and did not observe a particular enrichment of DNA damage-related proteins in our experiments.

In principle, the approach we describe here should be applicable to a wide range of cell types and cell states, which in turn may help reveal specific differences in G4 interactomes and biology. During the revision of this article, we became aware of an independent study that involved a pyrrolidine derivative of PDS^50^ and reported the identification of G4-related proteins in human SV589 and MM231 cells^51^. Although we noted some overlap between the studies (61 shared protein candidates), which somewhat validates the independent approaches, most of the G4-associated proteins identified by our CMPP approach were not found in the independent study. The different outcomes may have arisen due to variations in protein expression levels, chromatin states and G4 biology between the different cell lines. There were also some important technical differences between the two studies, which may have contributed to differences in the outcomes. In our study, we fractionated the nuclear proteins to focus on chromatin-associated proteins involved in G4 biology, and also to minimize the masking of physiologically relevant DNA G4 interactors by high-abundance, cytosolic RNA-binding proteins (for example, ribosomal proteins and elongation factors)^52^. In addition, we employed the diazirine crosslinker control 3, which lacks a G4 binding moiety to account for and factor out background binding (Methods), as considerable off-target binding to diazirine photocrosslinkers has been reported previously^37,53^.

Overall, our chemical method shows that it can provide an unbiased strategy for the global mapping of interacting proteins of nucleic acid structural features in live cells. Although this study focused on DNA G4 interactors, we also identified several candidates that are annotated as RNA-binding proteins. PDS can bind both DNA and RNA G4s with comparable affinity^43^ and, therefore, some of the identified proteins might, in principle, bind to nuclear RNA G4s. We envisage that future studies with RNA G4-specific probes^49^ might employ a similar approach to explore endogenous RNA G4-protein interactions. We also envision that the general principle will enable further studies to map endogenous interactomes of other nucleic acid structural features.

## Methods

Detailed synthetic procedures and full characterization of photoPDS-1 (1) and photoPDS-2 (2), biophysical assays and more detailed methods as well as general information are described in the Supplementary Information.

### Cell culture

Human embryonic kidney HEK293T cells (ATCC, CRL-3216) were grown in high-glucose DMEM (l-glutamine and pyruvate plus, GIBCO) supplemented with 10% (v/v) heat-inactivated fetal bovine serum (FBS). Human chronic myelogenous leukaemia K562 cells (ATCC, CCL-243) were cultured in RPMI1640 (Glutamine plus, Life Technologies) supplemented with 10% FBS (Life Technologies). Both cell lines were grown at 37 °C in a 5% CO_2_ atmosphere. Cells used in the experiments were passaged at least twice after being thawed. Cells were tested periodically for mycoplasma contamination.

### Co-binding-mediated proximity labelling of BG4

G4 Myc (7.3 µM) and the single-stranded mutated oligonucleotides were annealed in 10 mM Tris, pH 7.4, 200 mM KCl and ds Myc in 10 mM Tris, pH 7.4, 200 mM NaCl. The G4-specific antibody BG4^17^ (5 µl of 6.6 µM in PBS) was then incubated with 5 µl of annealed oligonucleotides at room temperature by gently shaking for 1 h, followed by adding 5 µl of the indicated probes in 10 mM Tris HCl, pH 7.4, 100 mM KCl and incubated at room temperature for another hour. The solution was directly irradiated under 365 nm light on ice for 10 min, and 1.7 µl of the ‘click’ mixture (2 μl of 50 mM CuSO_4_ in H_2_O, 2 μl of 50 mM TCEP (tris(2-carboxyethyl)phosphine) in H_2_O, 1 μl of 10 mM TAMRA-azide in DMSO and 5 μl of 2 mM TBTA (tris((1-benzyl-1H-1,2,3-triazol-4-yl)methyl)amine) in 1/4 DMSO/*t*-BuOH) was added and the mixture was gently shaken at room temperature for 1 h. Next, 5.6 µl of LDS loading buffer (4×) was added and the solution was heated at 70 °C for 10 min. Each sample (~22 μl) was loaded and separated by SDS-PAGE (NuPAGE 4 to 12% and Bis-Tris, 1.0 mm), visualized on a Bio-Rad ChemiDoc MP system and the obtained images processed using Image Lab (version 6.1.0) software. Three biological replicates were performed.

### Proximity labelling of G4 interactomes in live cells

The protocol was adapted from that described previously^13^. For gel-based experiments, HEK293T cells were grown in 6 cm dishes to a ~90% confluence at the time of treatment. Cells were carefully washed with 5 ml of Dulbecco’s phosphate-buffered saline (DPBS) (GIBCO) and then incubated with the indicated probe-containing fresh FBS-free DMEM media (2.5 ml) at 37 °C for 1 h, followed by direct irradiation under 365 nm light (UVP CL-1000 Ultraviolet Crosslinker, Fisher Scientific) on ice for 10 min. To harvest cells in cold DPBS (3 ml) they were scraped, centrifuged (300*g*, 5 min, 4 °C) and then washed with cold DPBS twice. Cell pellets were either treated directly or kept frozen at –80 °C until use. For MS-based experiments, a similar protocol as that above was used with minor modifications, which included that HEK293T cells were grown in 15 cm dishes to 80–90% confluence and then treated with 15 cm fresh FBS-free media that contained the indicated probes.

### Nuclear protein extraction for gel- and MS-based analysis

The cell pellets for 6 cm and 15 cm dishes were gently resuspended in 250 μl and 2.25 ml, respectively, of Hypotonic Buffer (10 mM HEPES, pH 7.4, 10 mM KCl and 1.5 mM MgCl_2_) with a protease inhibitor cocktail (PIC) (ThermoFisher, catalogue no. 78438) by pipetting several times and swelled on ice for 15 min. NP-40 (10%, 12.5 and 112.5 μl, respectively) was added and the pellets were vortexed at the highest setting for 10 s, centrifuged (900*g*, 10 min, 4 °C) to afford the nuclear pellets, which were then washed once with Hypotonic Buffer (250 μl and 1.5 ml, respectively). The isolated nuclear pellets were lysed in 50 and 250 μl, respectively, of high-salt Hypotonic Buffer (10 mM HEPES, pH 7.4, 400 mM NaCl, 10 mM KCl and 1.5 mM MgCl_2_) that contained PIC, 0.5% NP-40 and 2 mM phenylmethylsulfonyl fluoride, followed by adding 0.25 and 1.25 μl, respectively, of benzonase (Sigma-Aldrich, catalogue no. E1014) and incubating on ice for 30 min with vortexing at 10 min intervals. The lysates were centrifuged (16,000*g*, 10 min, 4 °C) to give the supernatant that contained nuclear proteome, which was transferred to a clean protein LoBind tube, and the protein concentration was determined by a BCA (bicinchoninic acid) protein assay.

### Gel-based analysis of probe-labelled nuclear G4 interactomes

Nuclear proteins (100 μg) were diluted with 50 mM HEPES, pH 7.4, to 80 μl in a clean 1.5 ml microcentrifuge tube. To dissolve the proteins, 10 μl of 4% SDS 50 mM HEPES, pH 7.4, was added, followed by adding 10 μl of a freshly prepared click mixture (2 μl of 50 mM CuSO_4_ in H_2_O, 2 μl of 50 mM TCEP in H_2_O, 1 μl of 10 mM TAMRA-azide in DMSO and 5 μl of 2 mM TBTA in 1/4 DMSO/*t*-BuOH). The mixture was gently shaken at room temperature for 1 h, followed by adding prechilled methanol (400 μl) and keeping it at –20 °C overnight. The precipitated protein pellets were collected by centrifuge (16,000*g*, 10 min, 4 °C) and washed with prechilled methanol (400 μl). After drying the pellets at room temperature for 5 min, 50 μl of a 1× LDS sample buffer that contained 2.5% v/v 2-mercaptoethonal was added and the solution was heated at 95 °C for 10 min. The sample (20 μ) was loaded per gel lane for SDS-PAGE (NuPAGE 4 to 12% and Bis-Tris, 1.0 mm) analysis, visualized by in-gel fluorescence scanning on a Bio-Rad ChemiDoc MP system. Three biological replicates for each experiment were performed.

### Enrichment of probe-labelled nuclear G4 interactomes for MS-based analysis

Nuclear proteins (700 μg) were diluted with 50 mM HEPES to 560 μl in a clean 5 ml microcentrifuge tube, to which 70 μl of 4% SDS 50 mM HEPES, pH 7.4, was added followed by 70 μl of a freshly prepared click mixture (14 μl of 50 mM CuSO_4_ in H_2_O, 14 μl of 50 mM TCEP in H_2_O, 7 μl of 10 mM Biotin-PEG_3_-azide in DMSO and 35 μl of 2 mM TBTA in 1/4 DMSO/*t*-BuOH). The mixture was incubated by rotating at room temperature for 1 h, followed by adding prechilled methanol (2.8 ml) and then left at –20 °C overnight for protein precipitation. The solution was centrifuged (16,000*g*, 10 min, 4 °C) and the obtained protein pellets were washed with prechilled methanol (2.8 ml 2×). After drying at room temperature for 5 min, the nuclear proteins were redissolved in freshly prepared 0.2% SDS urea (625 μl, 6 M in DPBS) by sonication. The protein solution was then transferred to a 2 ml Protein Lobind microcentrifuge tube, followed by adding 62.5 μl of a 1:1 mixture of TCEP (200 mM in DPBS) and potassium carbonate (600 mM in DPBS), and the mixture was incubated at 37 °C for 30 min to reduce the disulfides. Alkylation of the free thiols was performed by adding 87.5 μl of iodoacetamide (400 mM in DPBS) and the mixture was incubated at room temperature for 30 min in the dark. Then, 25 μl of 10% SDS in DPBS was added, followed by adding DPBS (1,075 μl) to dilute the solution to 0.2% SDS, and the solution was incubated with 100 μl of streptavidin magnetic beads (Dynabeads, MyOne, Streptavidin C1, Invitrogen, catalogue no. 65002), prewashed with DPBS (1.5 ml 3×), at room temperature for 1 h with gentle rotation. The magnetic beads were then sequentially washed (changing tubes between each washing buffer and every single Tris and ammonium bicarbonate wash) with 2% SDS in H_2_O at room temperature (2 ml 2×, one for 5 min and the other for 10 min), washing buffer 1 (0.1% sodium deoxycholate, 1% Triton X-100, 500 mM NaCl, 1 mM EDTA and 50 mM 4-(2-hydroxyethyl)-1-piperazineethanesulfonic acid, pH 7.5) at 4 °C (2 ml 2×, 5 min each), washing buffer 2 (250 mM LiCl, 0.5% NP-40, 0.5% sodium deoxycholate, 1 mM EDTA and 10 mM Tris, pH 8.0) at 4 °C (2 ml 2×, 5 min each), 50 mM Tris (2 ml 2×) and freshly prepared cold 100 mM NH_4_HCO_3_ in H_2_O (400 μl 2×). Beads were either treated directly or kept frozen at –20 °C until use.

### Label-free quantitative proteomics data analysis

The label-free experiment consisted of 24 samples distributed in 6 groups, which included the treatments with the G4-ligand probes 1 and 2 and the negative control probe 3. Missing values for 3 are imputed by replacing them with the minimum value, whereas those for 1 and 2 are imputed using the nearest neighbour method after removing peptides missing in more than half of samples in each group. The peptide intensities of the filtered peptides were analysed using the Bioconductor library qPLEXanalyzer^54^. To find differentially expressed proteins, a statistical analysis was carried out using the Bioconductor library limma^55^. Visualization of the results was performed with volcano plots and Venn diagrams using the R libraries ggplot2 (https://cran.r-project.org/web/packages/ggplot2/index.html), ggrepel (https://cran.r-project.org/web/packages/ggrepel/index.html) and VennDiagram (https://cran.r-project.org/web/packages/VennDiagram/index.html). UniprotKB keywords of differentially expressed proteins were extracted using the Retrieve/ID mapping online functionality^56^. The list of 79 G4-associated proteins in humans was downloaded from G4IPDB^41^ (accessed 20th November, 2020). The code is available on the github page dedicated to this study, https://github.com/sblab-bioinformatics/cmpp

### G4 affinity enrichment and western analysis

HEK293T cells were grown to ~80% confluence at the time of treatment. Cell pellets were swelled at a density of 10 million cells per 300 µl in a low salt buffer (20 mM HEPES, pH 7.4, 10 mM NaCl, 3 mM MgCl_2_, 0.2 mM EDTA and 1 mM dichlorodiphenyltrichloroethane (DTT)) that contained PIC on ice for 15 min. Then, 15 μl of 10% NP-40 was added and pellets were vortexed for 1 min, centrifuged (900 g, 10 min, 4 °C) to afford the nuclear pellets, which were then washed with low salt buffer. The nuclear pellets were lysed at a density of 30 million cells per 250 µl in high salt buffer (20 mM HEPES, pH 7.4, 500 mM NaCl, 3 mM MgCl_2_, 0.2 mM EDTA, 0.5% NP-40 and 1 mM DTT) that contained PIC by sonicating in a Diagenode Bioruptor Plus (ten cycles, 30 s on and 30 s off at each high setting, 4 °C). The lysates were centrifuged (16,000*g*, 10 min, 4 °C) to afford the nuclear proteins, and the concentration was measured using the BCA protein assay.

A slurry (50 µl) of Streptavidin MagneSphere paramagnetic beads (Promega, catalogue no. Z5481) was prewashed with pull-down buffer (25 mM HEPES, 10.5 mM NaCl, 110 mM KCl, 1 mM MgCl_2_, 0.01 mM ZnCl_2_, 20% v/v glycerol, 0.1% Igepal C-630, 1 mM DTT and PIC) that contained 3% bovine serum albumin (BSA) and 0.2 g l^–1^ salmon sperm DNA (Invitrogen, catalogue no. 15632011) three times (2 ml), and then 75 µg of nuclear proteins was added into 500 μl of pull-down buffer that contained 3% BSA and 0.2 g l^–1^ salmon sperm DNA, and precleared by incubating with the prewashed beads at 4 °C for 2 h. Meanwhile, another 50 µl of beads was washed in the same manner as above. Then, 50 µl of 10 µM annealed biotinylated oligonucleotides (Sigma-Aldrich) was added into 500 µl of pull-down buffer and incubated with the prewashed beads by rotation at room temperature for 30 min. The oligonucleotide immobilized beads were then washed with pull-down buffer (2 m 3×) and incubated with the precleared lysates (500 µl) by rotation at 4 °C overnight. The beads were washed with cold pull-down buffer (500 µl 5×) and the biotinylated oligonucleotides on the beads were eluted in 25 µl of LDS sample buffer that contained freshly prepared 50 mM DTT by heating at 70 °C for 10 min. Next, 3 µl of the LDS sample buffer were analysed with capillary electrophoresis in a Wes Simple Western system (ProteinSimple) according to the instructions of the manufacturer, or samples were kept frozen at –20 °C until analysis. The primary antibodies (Supplementary Table 4) and the corresponding secondary antibodies (anti-rabbit) were used to detect the target signal bands, which were analysed by the software Compass for SW (ProteinSimple).

### Enzyme-linked immunosorbent assay

ELISAs for binding affinity and specificity were performed as described previously^17^ with minor modifications. Briefly, biotinylated oligonucleotides were bound to Pierce streptavidin-coated high capacity plates (ThermoFisher) followed by blocking with 3% BSA and incubation with full-length recombinant human GST-tagged UHRF1 (Abnova, catalogue no. H00029128-P01) and DDX24 (Abnova, catalogue no. H00057062-P01), HIS-tagged SMARCA4 (Abcam, catalogue no. ab82237), RBM22 (OriGene, TP760056) and Myc/DDK-tagged DDX1 (OriGene, TP308769) in ELISA buffer (100 mM KCl and 50 mM KH_2_PO_4_, pH 7.4). After three washes with the ELISA buffer, detection was achieved with an anti-GST HRP (horseradish peroxidase)-conjugated antibody (Abcam, catalogue no. ab3416) diluted to 1:5,000, anti-FLAG HRP-conjugated antibody (Abcam, ab1238,) diluted to 1:15,000 or anti-HIS HRP-conjugated antibody (BioLegend, catalogue no. 652503) diluted to 1:3,000 in an ELISA buffer that contained 3% BSA and 3,3′,5,5′-tetramethylbenzidine ELISA substrate (slow kinetic rate) (Abcam, ab171525). Signal intensity was measured at 450 nm on a SPECTROstar nano microplate reader (BMG Labtech). *K*_d_ values were calculated from binding curves assuming a one-site binding model in GraphPad Prism, and standard error of means from three replicates are reported.

### SMARCA4 ChIP-seq

SMARCA4 ChIP-seq was performed essentially as described previously^57^. Briefly, cells were first crosslinked in 2 mM disuccinimidyl glutarate (ThermoFisher) in PBS for 30 min and then in 1% formaldehyde in the medium for 10 min at room temperature. The cells were quenched with 0.125 M glycine for 5 min and washed twice in ice-cold PBS. Chromatin was isolated and prepared using a ChIP-qPCR Kit (Chromatrap) and sonicated using a Bioruptor Plus (Diagenode) to an average DNA size of 150–400 base pairs. Magnetic protein G Dynabeads (ThermoFisher) were washed with PBS that contained 1% w/v BSA (Sigma-Aldrich), incubated with 5 µg of ChIP-grade antibody against SMARCA4 (Abcam, ab110641) for 1 h at room temperature and washed five times with PBS that contained 1% w/v BSA. Solubilized chromatin from 5 × 10^6^ cells was immunoprecipitated with antibody conjugated beads in RIPA buffer (50 mM Tris pH 7.4, 150 mM NaCl, 1% Igepal CA-630 and 0.5% sodium deoxycholate) for 12 h at 4 °C. Magnetic beads were washed 5× with RIPA buffer and chromatin was eluted. After crosslinking reversal, RNAase A (Ambion) and proteinase K (ThermoFisher) treatment, ChIP DNA was extracted using a Min-Elute purification kit (Qiagen). Sequencing libraries of ChIP DNA and input controls were generated using the NEBNext Ultra DNA Library Prep Kit for Illumina (NE Biolabs) following the manufacturer’s protocol.

### SMARCA4 ChIP-seq data analysis

Bioinformatics data analyses and processing were performed using Bash, R and Python programming languages. The following tools were also used: cutadapt (version 1.16)^58^, BWA (v0.7.15)^59^, Picard (v2.14.0; http://broadinstitute.github.io/picard), MACS2 (v2.1.1)^60^, bedtools^61^ (v2.26.0), SAMtools (v1.6)^62^, deepTools (v3.1.2)^63^ and Intervene (v0.6.4)^64^. Code is available in the github page dedicated to this study, https://github.com/sblab-bioinformatics/cmpp. Raw fastq files were trimmed with cutadapt^58^ to remove adapter sequences and low-quality reads (mapping quality <10). Reads were aligned to the human reference genome (version hg19) with BWA^59^ and duplicates marked using Picard (v 2.14.0; http://broadinstitute.github.io/picard) and removed using SAMtools^62^. G4 ChIP and SMARCA4 ChIP peaks were called by MACS2^60^ (q-value < 0.05). Peak overlaps in different replicates were visualized with Intervene^64^. Peaks were merged from replicates with bedtools^61^ and high confidence peaks were defined as those overlapping in two out of three replicates (SMARCA4) or five out of eight replicates (G4 ChIP-seq) as described previously^21^. Fragment coverage bigWig files were computed at a 50 base pair resolution, 200 base pair average fragment size and normalization to sequencing depth (RPKM) using deepTools^63^. Signal distribution from the SMARCA4 ChIP in K562 G4 ChIP-seq peaks and potential G4s was computed using the plotProfile function in deepTools^63^.

### Reporting Summary

Further information on research design is available in the Nature Research Reporting Summary linked to this article.

## Online content

Any methods, additional references, Nature Research reporting summaries, source data, extended data, supplementary information, acknowledgements, peer review information; details of author contributions and competing interests; and statements of data and code availability are available at 10.1038/s41557-021-00736-9.

## Supplementary information

Supplementary information Supplementary Tables 1–6, synthetic procedures, NMR spectra, other experimental methods and additional information.

Reporting Summary

Supplementary Table 1 The label-free quantitative proteomics data reported in this study.

## Data Availability

The label-free quantitative proteomics data reported in this study are included in Supplementary_Dataset_CMPP, which contains peptide intensities, metadata and enriched proteins from the 1 versus 3 and 2 versus 3 statistical comparisons. The SMARCA4 ChIP-seq data have been deposited in the NCBI GEO repository under accession number GSE165124. The BG4 ChIP-seq data were generated in a previous study^21^ and are available under accession number GSE107690. Source data are provided with this paper.

## photoPDS-1

PubChemID: 441286341
InChIKey: GMYWHLRQNFJVCU-UHFFFAOYSA-N
MDL Molfile
Chemdraw file

## photoPDS-2

PubChemID: 441286342
InChIKey: AOBFHOVZGBCROA-UHFFFAOYSA-N
MDL Molfile
Chemdraw file

## 3-(3-(but-3-yn-1-yl)-3*H*-diazirin-3-yl)-*N*-methylpropanamide

PubChemID: 441286332
InChIKey: AXQJCYNFLNSQDW-UHFFFAOYSA-N
MDL Molfile
Chemdraw file

## dimethyl 4-hydroxypyridine-2,6-dicarboxylate

PubChemID: 441286333
InChIKey: FERASHUCDLDGHK-UHFFFAOYSA-N
MDL Molfile
Chemdraw file

## 4-(2-(((benzyloxy)carbonyl)amino)ethoxy)pyridine-2,6-dicarboxylic acid

PubChemID: 441286334
InChIKey: MZNZWTLTAMFOAM-UHFFFAOYSA-N
MDL Molfile
Chemdraw file

## *tert*-butyl (2-((2-aminoquinolin-4-yl)oxy)ethyl)carbamate

PubChemID: 441286335
InChIKey: QZMRBSSSRBBFCU-UHFFFAOYSA-N
MDL Molfile
Chemdraw file

## di-*tert*-butyl (((((4-(2-(((benzyloxy)carbonyl)amino)ethoxy)pyridine-2,6-dicarbonyl)bis(azanediyl))bis(quinoline-2,4-diyl))bis(oxy))bis(ethane-2,1-diyl))dicarbamate

PubChemID: 441286336
InChIKey: YZEHAOMWRTUTER-UHFFFAOYSA-N
MDL Molfile
Chemdraw file

## di-*tert*-butyl (((((4-(2-aminoethoxy)pyridine-2,6-dicarbonyl)bis(azanediyl))bis(quinoline-2,4-diyl))bis(oxy))bis(ethane-2,1-diyl))dicarbamate

PubChemID: 441286337
InChIKey: MJTXICPYQLONGT-UHFFFAOYSA-N
MDL Molfile
Chemdraw file

## 3-(but-3-yn-1-yl)-3-(2-iodoethyl)-3*H*-diazirine

PubChemID: 441286338
InChIKey: XKVWLCIADFSFAP-UHFFFAOYSA-N
MDL Molfile
Chemdraw file

## 3-(3-(but-3-yn-1-yl)-3*H*-diazirin-3-yl)-*N*-(2-(2-(2-iodoethoxy)ethoxy)ethyl)propanamide

PubChemID: 441286339
InChIKey: CZIDYZGWDNQMMS-UHFFFAOYSA-N
MDL Molfile
Chemdraw file

## 3-(3-(but-3-yn-1-yl)-3*H*-diazirin-3-yl)propanoic acid

PubChemID: 441286340
InChIKey: JRNSKSYASHYNCM-UHFFFAOYSA-N
MDL Molfile
Chemdraw file

## Source data

Source Data Fig. 2 Uncropped gels and statistical source data. Source Data Fig. 3 Uncropped gels and statistical source data. Source Data Fig. 4 Uncropped Western Blots and statistical source data. Source Data Fig. 5 Statistical Source Data. Source Data Extended Data Fig. 1 Uncropped gels and statistical source data. Source Data Extended Data Fig. 2 Uncropped gels and statistical source data. Source Data Extended Data Fig. 3 Statistical Source Data. Source Data Extended Data Fig. 4 Uncropped Western Blots and statistical source data.

## Code availability

For details about the bioinformatics data analysis, see https://github.com/sblab-bioinformatics/cmpp
