# Supplementary material for: Chemical profiling of DNA G-quadruplex-interacting proteins in live cells
Source: Nat Chem. 2021 Jun 28;13(7):626–33. doi: 10.1038/s41557-021-00736-9 (PMC8245323; doi:10.1038/s41557-021-00736-9)
Supplement: Supplementary file 1 — Supplementary Tables 1–6, synthetic procedures, NMR spectra, other experimental methods and additional information. [file 41557_2021_736_MOESM23_ESM.pdf]

nature portfolio
https://doi.org/10.1038/s41557-021-00736-9

Supplementary information

Chemical profiling of DNA G-quadruplex-interacting proteins in live cells

In the format provided by the authors and unedited

Supplementary Information

Chemical profiling of DNA G-quadruplex-interacting proteins in live cells

Xiaoyun Zhang[1,5], Jochen Spiegel[2,5], Sergio Martínez Cuesta[1,2,4], Santosh Adhikari[1], Shankar Balasubramanian[1,2,3*]

[1]Department of Chemistry, University of Cambridge, Cambridge, UK.
[2]Cancer Research UK Cambridge Institute, Li Ka Shing Centre, University of Cambridge, Cambridge, UK.
[3]School of Clinical Medicine, University of Cambridge, Cambridge, UK.
[4]Current address: Data Sciences and Quantitative Biology, Discovery Sciences, AstraZeneca, Cambridge, UK
[5]These authors contributed equally: Xiaoyun Zhang, Jochen Spiegel

Correspondence to: sb10031@cam.ac.uk.

Table of contents

General information 2
Experimental methods 2
Supplementary Table 1 5
Supplementary Table 2 6
Supplementary Table 3 7
Supplementary Table 4 8
Supplementary Table 5 9
Supplementary Table 6 10
Synthetic procedures 11
NMR spectra 15
References 21

General information

All chemicals and reagents were purchased from Sigma-Aldrich, Alfa Aesar and Fisher Scientific. All organic solvents were distilled by standard purification methods before use or purchased as anhydrous from Sigma-Aldrich. All reactions were performed in oven-dried glassware under argon unless otherwise stated. NMR spectra were recorded on a Bruker 400 MHz Avance III HD Spectrometer or a 500 MHz DCH Cryoprobe Spectrometer operating at 400 and 500 MHz for ^{1}H NMR, and at 100 and 125 MHz for ^{13}C NMR respectively in the indicated solvents. NMR data are reported as follows: chemical shifts in parts per million (ppm) referring to the solvent residual peak, multiplicities (s = singlet, d = doublet, t = triplet, q = quartet; m = multiplet, br = broad) and coupling constant values in Hz. LC-MS was performed on an Amazon ESI-MS (Bruker) connected to a Dionex UltiMate 3000 UHPLC system (Thermo Fisher Scientific). High-resolution mass spectra (HRMS) were obtained from a Waters LCT Premier (ESI) spectrometer. Flash column chromatography was performed using CombiFlash Rf (Teledyne ISCO) with silica gel puriFlash columns (Interchim). Chelidamic acid dimethyl ester (**1**),[1] compound **S3**[2] and pyridostatin (PDS)[2] were prepared according to the previously reported procedures. 3-(but-3-yn-1-yl)-3-(2-iodoethyl)-3h-diazirine (**6**) and 3-[3-(but-3-yn-1-yl)-3H-diazirin-3-yl]propanoic acid (**8**) were purchased from Fluorochem and Sigma-Aldrich, respectively.

Experimental methods

Circular dichroism spectroscopy. Circular dichroism (CD) spectra were obtained on an Applied Photo-physics Chirascan circular dichroism spectropolarimeter. Measurements were taken in 1 mm path length quartz cuvettes over a range of 200 – 330 nm at 25 °C using time-per-point of 0.5 s at 1 nm intervals and 1 nm bandwidth. The recorded spectra represent a smoothed average of three scans, baseline corrected for the indicated buffer used (molar ellipticity θ is quoted in 105° cm^2 $dmol^{-1}$).

Oligonucleotide annealing. HPLC purified oligonucleotides were purchased from Sigma-Aldrich unless otherwise stated. For G4s or the single-strand mutants, the indicated concentration of oligonucleotides in 10 mM Tris HCl, pH 7.4, 100 mM KCl were annealed at 95 °C for 5 min followed by gradually cooling to 20 °C. For double-stranded DNA, forward and reverse strand oligonucleotides were mixed by 1:1 and annealed in 10 mM Tris HCl, pH 7.4, 100 mM NaCl in the same way.

FRET melting assay. Oligonucleotides (see Supplementary Table 1) were purchased from Biomers. 400 nM oligonucleotides were annealed in FRET buffer (60 mM potassium cacodylate, pH = 7.4) at 95 °C for 5 min followed by gradually cooling to 20 °C. A series of probe concentrations were prepared in a 96-well plate: 150 μL of 6 μM ligand in FRET buffer was prepared as the initial concentration. Subsequent serial dilutions were made by adding 100 μL of probe solutions to 50 μL of FRET buffer, resulting in 12 concentrations including a no-probe control. 25 μL per solutions were transferred to another 96-well plate, followed by adding 25 μL of annealed oligonucleotide solutions to each well. The plate was then sealed with an adhesive transparent cover and shaken gently for 10 min. Measurements of restoring

FAM signal were recorded on a Bio-Rad CFX96 Touch Real-Time PCR Detection System by ramping from 25°C to 95 °C at 0.5 °C/min. Melting temperatures were determined by the first-derivative maxima of relative fluorescence unit (RFU) value against time, and ΔT_m was calculated by baseline correction of melting temperatures subtracting no-probe control. A one-site binding model in GraphPad Prism 7 was utilised to fit FRET curves. Mean was calculated from two replicates.

Fluorescence quench binding assay. The protocol was adapted from that described previously.[3] Oligonucleotides (Supplementary Table 2) were purchased from Biomers. 10 nM oligonucleotides in assay buffer (50 mM Tris HCl, pH 7.2, 150 mM KCl, 0.5 w/v % CHAPS, 0.05 v/v % Triton X-100) were annealed. A series of probe dilutions were prepared in a 96-well plate: 100 μL of 50 μM probe in 0.1 v/v % DMSO in water was prepared as the initial concentration. Subsequent serial dilutions were made by adding 50 μL of ligand solutions to 50 μL of 0.1 v/v % DMSO in water, resulting in a total of 12 concentrations including a no-probe control. 10 μL per solution of the series of dilutions were transferred to another 96-well plate, followed by adding 90 μL of the annealed oligonucleotide solution to each well to afford the final concentration of 9 nM. The plate was sealed with an adhesive foil cover and gently shaken for 2 h. End-point fluorescence was then measured on a fluorescence plate reader (BMG PHERAstar Plus). Differences in RFU were converted to absolute values relative to the no-probe control. The range of observed absolute RFU values were normalized as fraction bound. Dissociation constants (K_d) were calculated assuming a one-site binding model using GraphPad Prism 7. Standard deviations (S.D.) were calculated from four replicates.

Cell viability assay. Cells were seeded in a 96-well flat-bottom plate at a density of 20,000 cells/well in 100 μL of DMEM media and allowed to grow at 37 °C for 20 h. Given that cells were incubated with probes for 60 min in both gel-based and MS-based experiments, cells for viability assay were treated with probes for 75 min in triplicates. Cell viability was measured using the CellTiter-Glo Luminescent Cell Viability Assay (Promega). End-point luminescence was then measured on a multi-mode plate reader (BMG PHERAstar Plus). A zero-correction was done by subtracting the media absorbance, and the corrected absorbance was converted to cell viability (%) relative to the no-ligand control. Dose-dependent cell viability was calculated using GraphPad Prism 7. Standard error of means (s.e.m.) were calculated from four replicates.

HPLC-MS/MS analysis. To digest bead-bound proteins, 100 ng of trypsin in 100 mM NH_4HCO_3 was directly added onto the NH_4HCO_3 washed beads. The samples were vortexed for 15 s in every 2–3 min for the first 15 min to ensure that the beads are evenly suspended in the trypsin solution, followed by digestion overnight at 37 °C in an oven incubator without further agitation of the beads. 100 ng of additional trypsin was then added to each sample, and the samples were digested for another 4 h at 37 °C. The supernatant was transferred into a clean tube and 5% formic acid was added to make the final concentration of formic acid to 0.5% (v/v) to stop the digestion reaction.

For desalting the digested samples, the C18 cartridge was first conditioned with 50% (v/v) acetonitrile/water (100 μL × 2), equilibrated with 0.1% (v/v) formic acid (100 μL × 2), and the acidified peptides were loaded onto the cartridge. The peptide-loaded cartridge was

washed three times with 0.1% (v/v) formic acid, and the peptides were then eluted three times with 100 µL of 60% (v/v) acetonitrile/0.1% (v/v) formic acid. Eluates were combined and then dried completely by vacuum centrifugation.

Each dried peptide sample was reconstituted in 10 µL of 0.1% (v/v) formic acid, and 9 µL was injected in the LC for the analysis. Peptides were loaded and separated on a reverse-phase trap column 2 cm (100 µm i.d.) and analytical column (25 cm × 75 µm i.d.) respectively with 5–45% acetonitrile gradient in 0.1% formic acid and 5% DMSO at 300 nL/min flow rate. In each data collection cycle, one full MS scan (400–1,600 m/z) was acquired in the Orbitrap (60 K resolution, automatic gain control (AGC) setting of 3×10^6 and Maximum Injection Time (MIT) of 100 ms). The most abundant ions with a top 10 settings were selected for fragmentation by collision induced dissociation (CID). CID was performed with a collision energy of 28%, an AGC setting of 2×10^4, an isolation window of 2.0 Da, a MIT of 100ms. Previously analysed precursor ions were dynamically excluded for 25s.

The Proteome Discoverer 2.2 (Thermo Scientific) was used for the processing of CID tandem mass spectra. The SequestHT search engine was used and all the spectra searched against the Uniprot Homo sapiens FASTA database (taxon ID 9606). All searches were performed including Methionine oxidation (+15.9949Da) and Deamidation on Asparagine and Glutamine (+0.984) as dynamic modifications. Mass spectra were searched using precursor ion tolerance 20 ppm and fragment ion tolerance 0.02 Da. For peptide confidence, 1% FDR was applied and peptides uniquely matched to a protein were used for precursor quantification using minora and for further analysis.

Supplementary Table 1. DNA oligonucleotides for FRET melting assay

Oligomer	Sequence (5' to 3')	Source	Ref
G4 Kit1	FAM-**GGG**A**GGG**CGCT**GGG**AGGA**GGG**-TAMRA	Biomers	(*4*)
G4 Myc	FAM-TGA**GGG**T**GGG**TA**GGG**T**GGG**TAA-TAMRA	Biomers	(*4*)
G4 Telo	FAM-**GGG**TTA**GGG**TTA**GGG**TTA**GGG**-TAMRA	Biomers	(*4*)
dsDNA	FAM-TATAGCTATA-HEG-TATAGCTATA-TAMRA	Biomers	(*4*)

FAM = fluorescein; TAMRA = tetramethylrhodamine; HEG = [(-CH_2CH_2O-)$_6$].

Supplementary Table 2. DNA oligonucleotides for fluorescence quench binding assay

Oligomer	Sequence (from 5' to 3')	Source	Ref
G4 Telo	**Cy5**-A**GGG**TTA**GGG**TTA**GGG**TTA**GGG**T	Biomers	(*3*)
G4 Kit1	**Cy5**-A**GGG**A**GGG**CGCT**GGG**AGGA**GGG**	Biomers	(*3*)
G4 Myc	T**GGGG**A**GGG**T**GGGG**A**GGG**T**GGGG**AAGG-**Cy5**	Biomers	(*3*)
dsDNA	**Cy5**-CAATCGGATCGAATTCGATCCGATTG	Biomers	(*3*)

Cy5 = cyanine 5.

Supplementary Table 3. DNA oligonucleotides for G4 pull-down

**Oligomer**	**Sequence (5’ to 3’)**	**Source**	**Ref**
G4 Myc	TGA**GGG**T**GGG**TA**GGG**T**GGG**TAATTTTT[TgBtn]	Sigma-Aldrich	(*5*)
G4 Kit1	A**GGG**A**GGG**CGCT**GGG**AGGA**GGG**TTTTT[TgBtn]	Sigma-Aldrich	(*6*)
G4 Kit2	C**GGG**C**GGG**CGCGA**GGG**A**GGG**TTTTTT[TgBtn]	Sigma-Aldrich	(*7*)
G4 TBA	[Btn]**GG**TT**GG**TGTGT**GG**TT**GG**	Sigma-Aldrich	(*8*)
G4 BCL2	**GGG**CGC**GGG**AGGAATT**GGG**C**GGG**TTTTT[TgBtn]	Sigma-Aldrich	(*9*)
ss mutMyc	TGA**GTG**T**GTG**TA**GTG**T**GTG**TAATTTTT[TgBtn]	Sigma-Aldrich	-
ds Myc	TGA**GGG**T**GGG**TA**GGG**T**GGG**TAATTTTT[TgBtn]<br>TTACCCACCCTACCCACCCTCA	Sigma-Aldrich	-
ss mutKit1	A**GTG**A**GTG**CGCT**GTG**AGGA**GTG**TTTTT[TgBtn]	Sigma-Aldrich	-
ds Kit1	A**GGG**A**GGG**CGCT**GGG**AGGA**GGG**TTTTT[TgBtn]<br>CCCTCCTCCCAGCGCCCTCCCT	Sigma-Aldrich	-
ss mutBCL2	**GTG**CGC**GTG**AGGAATT**GTG**C**GTG**TTTTT[TgBtn]	Sigma-Aldrich	-

Btn = biotin; Tg = tetraethylene glycol.

Supplementary Table 4. Antibodies for western blot and ChIP-seq analysis

Antibodies	Species	Source	Cat#
TTF2	rabbit	Proteintech	13722-1-AP
RBM22	rabbit	Proteintech	22103-1-AP
HMGB2	rabbit	Proteintech	14597-1-AP
SMARCA4	rabbit	abcam	ab110641
UHRF1	rabbit	abcam	ab194236
DDX1	rabbit	abcam	ab70252
DDX24	rabbit	abcam	ab70462

Supplementary Table 5. Western blot quantification of RBM22 (corresponding to Fig. 4)

**Oligomer**	**Peak area**		**Relative intensity [%][a]**
	**repeat 1**	**repeat 2**	
10% lysates	15485	30206	10.00
beads	5992	2601	1.880
**G4 Myc**	80153	85227	**36.20**
ss mutMyc	11145	18233	6.430
ds Myc	12086	9655	4.760
**G4 Kit1**	32818	60952	**20.52**
ss mutKit1	7608	5290	2.820
ds Kit1	1320	1872	0.700
**G4 Kit2**	63721	34370	**21.47**
G4 TBA	6289	3656	2.180
**G4 BCL2**	39301	29750	**15.11**
ss mutBCL2	2890	3578	1.420

[a] Peak area under the curve normalized to total lysate calculated based on the 10% input control. Mean was calculated from two independent experiments (n = 2).

Supplementary Table 6. Oligonucleotides for ELISA

Oligomer	Sequence (5' to 3')	Source	Ref
G4 Myc	[Btn]TGA**GGG**T**GGG**TA**GGG**T**GGG**TAA	Sigma-Aldrich	(*10*)
G4 Kit1	[Btn]A**GGG**A**GGG**CGCT**GGG**AGGA**GGG**	Sigma-Aldrich	(*10*)
G4 NRAS	[Btn]UGU**GGG**A**GGGG**C**GGG**UCU**GGG**UGC	Sigma-Aldrich	(*11*)
ss mutMyc	[Btn]TGA**GTG**T**GTG**TA**GTG**T**GTG**TAA	Sigma-Aldrich	-
ds Myc	[Btn]TGA**GGG**T**GGG**TA**GGG**T**GGG**TAA<br>TTACCCACCCTACCCACCCTCA	Sigma-Aldrich	-
ss mutKit1	A**GTG**A**GTG**CGCT**GTG**AGGA**GTG**	Sigma-Aldrich	-
ds Kit1	[Btn]A**GGG**A**GGG**CGCT**GGG**AGGA**GGG**<br>CCCTCCTCCCAGCGCCCTCCCT	Sigma-Aldrich	-
Hemi-methylated ds Kit1	[Btn]A**GGG**A**GGG**CGCT**GGG**AGGA**GGG**<br>CCCTCCTCCCAGXGCCCTCCCT	Sigma-Aldrich<br>Sigma-Aldrich	-
mutNRAS	[Btn]UGU**AGA**A**AGAG**C**AGA**UCU**AGA**UGC	Sigma-Aldrich	(*11*)

Btn = biotin; X = 5-methyldeoxycytosine.

Synthetic procedures

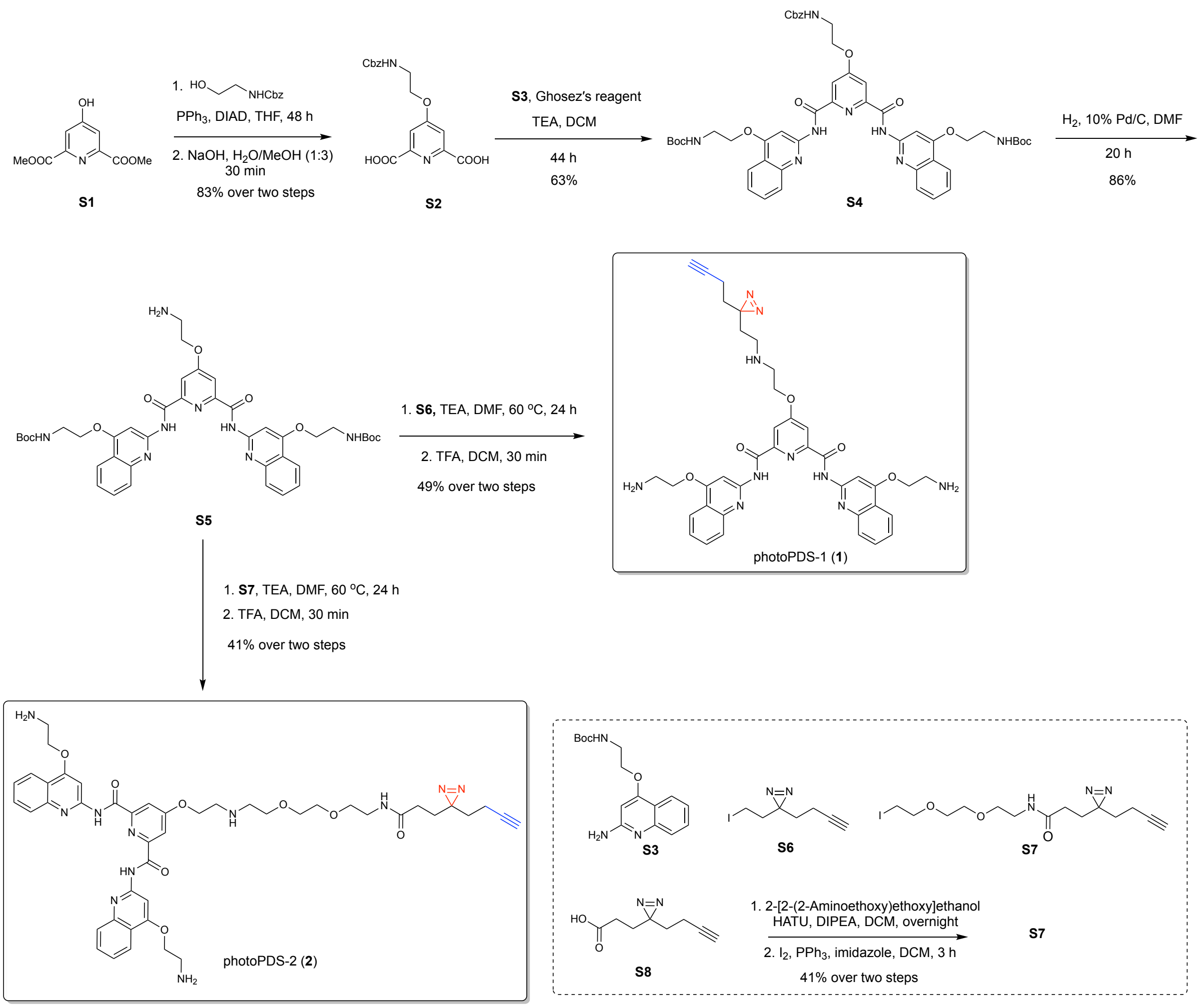

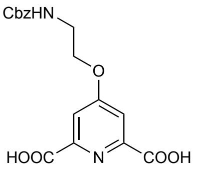

S2

4-(2-(((benzyloxy)carbonyl)amino)ethoxy)pyridine-2,6-dicarboxylic acid (S2): Chelidamic acid dimethyl ester **S1**[1] (634 mg, 3.0 mmol), benzyl *N*-(2-hydroxyethyl)carbamate (878 mg, 4.5 mmol) and triphenylphosphine (1.57 g, 6.0 mmol) were dissolved in 20 mL dry THF and the solution was cooled to 0 °C. DIAD (886 µL, 4.5 mmol) was added dropwise under argon. The reaction was allowed to warm to room temperature and stirred for 48 h. The solvent was removed in vacuo and the residue was purified by flash silica column chromatography (0% ~ 100% EtOAc in petroleum ether) to give the crude dimethyl ester product as a white solid which was used directly into next step without purification. The crude product was dissolved in 9 mL methanol and 3 mL 2 M NaOH solution was added. The mixture was stirred at room temperature for 30 min. The solution was concentrated in vacuo and then extracted with EtOAc (10 mL × 3). The aqueous layer was collected and adjusted to pH = 2 with 1 M HCl. The solution was then extracted with EtOAc (20 mL × 3). The organic layers were combined, washed with brine (5 mL), dried over anhydrous $MgSO_4$ and concentrated in vacuo to afford the title compound **S2** as a white solid (903 mg, 2.5 mmol, 83% yield over two steps): ^1H NMR (400 MHz DMSO-d_6): δ 7.70 (s, 2H), 7.50 (t, *J* = 5.6 Hz, 1H), 7.37 – 7.28 (m, 5H), 5.02 (s, 2H), 4.26 (t, *J* = 5.6 Hz, 2H), 3.42 (q, *J* = 5.6 Hz, 4H); ^{13}C NMR (100 MHz

DMSO-d_6): δ 166.6, 165.3, 156.3, 149.8, 137.1, 128.4, 127.82, 127.77, 113.7, 67.5, 65.4; HRMS (ESI-TOF): $[M+H]^+$ calculated for $C_{17}H_{17}N_2O_7$: 361.1036, found: 361.1024.

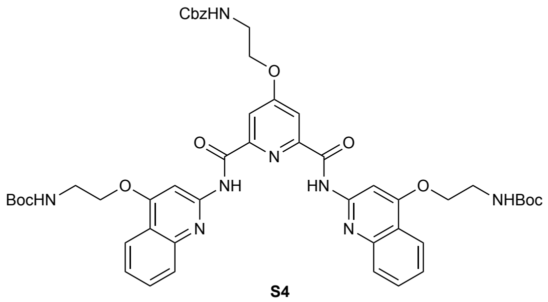

S4

Triprotected PDS (S4): Compound **S2** (412 mg, 1.1 mmol) was suspended in 15 mL dry CH_2Cl_2 and cooled to 0 °C under argon. Ghosez's reagent (380 µL, 2.82 mmol) was added slowly and the mixture was stirred at 0 °C for 1 h. Triethylamine (480 µL, 3.44 mmol) was added dropwise and the mixture was stirred for additional 30 min. **S3**[2] (866 mg, 2.85 mmol) was then added at 0 °C and the mixture was allowed to warmed to room temperature and stirred for 44 h. After filtration, the white precipitate was obtained, washed with acetonitrile and dried to give the title compound **S4** as a white solid (675 mg, 0.72 mmol, 63% yield): ^{1}H NMR (400 MHz DMSO-d_6): δ 12.03 (s, 2H), 8.24 (d, *J* = 8.0 Hz, 2H), 8.05 (s, 2H), 7.95 – 7.89 (m, 4H), 7.77 (t, *J* = 7.6 Hz, 2H), 7.57 (t, *J* = 6.4 Hz, 1H), 7.50 (t, *J* = 7.6 Hz, 2H), 7.37 – 7.27 (m, 5H), 7.19 (t, *J* = 6.0 Hz, 2H), 5.05 (s, 2H), 4.36 (t, *J* = 5.2 Hz, 2H), 4.27 (d, *J* = 5.2 Hz, 4H), 3.57 – 3.46 (m, 6H), 1.40 (s, 18H); ^{13}C NMR (125 MHz DMSO-d_6): δ 167.1, 163.2, 162.2, 156.3, 155.9, 152.4, 151.1, 147.0, 137.1, 130.6, 128.3, 127.80, 127.76, 126.8, 124.4, 122.2, 119.2, 112.1, 94.9, 77.9, 67.8, 67.5, 65.4, 28.2; HRMS (ESI-TOF): $[M+H]^+$ calculated for $C_{49}H_{55}N_8O_{11}$: 931.3990, found: 931.3971.

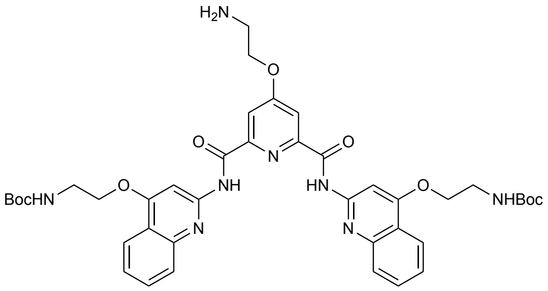

S5

Diprotected PDS (S5): Compound **S4** (300 mg, 0.32 mmol) was dissolved in 10 mL DMF. 10% Pd/C (30 mg) was then added and the H_2 balloon was placed over the reaction. The reaction was stirred at room temperature for 20 h. The balloon was removed and another 10 - 15 mL DMF was added to the mixture. The turbid reaction mixture was heated to 110-120 °C to completely dissolved the precipitated solid, filtered hot through a celite pad. The filtrate was concentrated in vacuo and the obtained solid was washed with CH_2Cl_2, acetonitrile and dried to give the title compound **S5** as a white solid (222 mg, 0.29 mmol, 86% yield): ^{1}H NMR (500 MHz DMSO-d_6): δ 8.23 (d, *J* = 8.5 Hz, 2H), 8.05 (s, 2H), 7.92 (d, *J* = 8.5 Hz, 2H), 7.91 (s, 2H), 7.76 (t, *J* = 7.5 Hz, 2H), 7.50 (t, *J* = 7.5 Hz, 2H), 7.21 (t, *J* = 6.0 Hz, 2H), 4.27 (t, *J* = 5.5 Hz, 4H), 4.23 (t, *J* = 5.5 Hz, 2H), 3.54 (q, *J* = 5.5 Hz, 4H), 2.96 (t, *J* = 6.0 Hz, 2H), 1.40 (s, 18H); ^{13}C NMR (125 MHz DMSO-d_6): δ 167.4, 163.2, 162.2, 155.9, 152.4, 151.0, 147.0, 130.6, 126.8, 124.4, 122.2, 119.2, 112.1, 94.9, 77.9, 71.4, 67.8, 40.5, 28.2; HRMS (ESI-TOF): $[M+H]^+$ calculated for $C_{41}H_{49}N_8O_9$: 797.3623, found: 797.3604.

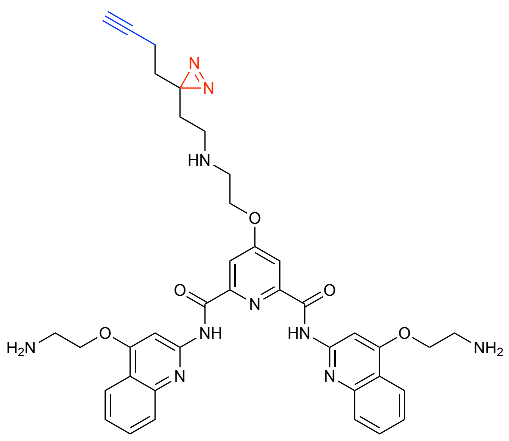

photoPDS-1 (**1**)

PhotoPDS-1 (1): S5 (52 mg, 65 μmol) was dissolved in 5 mL DMF. Next, 3-(but-3-yn-1-yl)-3-(2-iodoethyl)-3h-diazirine **S6** (17 mg, 0.0685 mmol) and triethylamine (28 μL, 0.200 mmol) were added and the reaction mixture was stirred at 60 °C for 24 h. The solvent was removed in vacuo and the crude product was used directly without further purification. The crude product was dissolved in 1 mL CH_2Cl_2 and 0.5 mL TFA was added. The mixture was stirred at room temperature for 30 min. The solvents were removed in vacuo and the residue was purified by HPLC (5% ~ 100% acetonitrile (with 0.1% TFA) in water (with 0.1% TFA) over 30 min). The most acetonitrile was removed in vacuo, and the rest of solvents were removed through freeze-drying to give the TFA salt of **1** as a white solid (23 mg, 32 μmol, 49% yield over two steps): ^{1}H NMR (400 MHz, Methanol-*d*$_4$) δ 8.49 (dd, *J* = 8.4 Hz, 0.8 Hz, 2H), 8.20 (s, 2H), 8.197 (s, 2H), 8.06 (d, *J* = 8.4 Hz, 2H), 7.93 (ddd, *J* = 8.4, 7.2 Hz, 1.6 Hz, 2H), 7.68 (ddd, *J* = 8.4 Hz, 7.2 Hz, 1.2 Hz, 2H), 4.75 (t, *J* = 4.8 Hz, 4H), 4.66 (t, *J* = 4.8, 2H), 3.68 (t, *J* = 4.8 Hz, 4H), 3.62 (t, *J* = 4.8 Hz, 2H), 3.16 – 3.10 (m, 2H), 2.36 (t, *J* = 2.8 Hz, 1H), 2.09 (td, *J* = 7.2 Hz, 2.8 Hz, 2H), 1.95 – 1.89 (m, 2H), 1.72 (t, *J* = 7.2 Hz, 2H); ^{13}C NMR (100 MHz, Methanol-*d*$_4$) δ 169.0, 167.0, 164.2, 152. 5, 151.2, 143.3, 134.3, 127.7, 124.3, 124.2, 120.2, 114.2, 95.3, 83.4, 70.8, 67.7, 65.9, 47.7, 44.1, 39.8, 32.8, 31.0, 27.0, 13.8; HRMS (ESI-TOF): $[M+H]^+$ calculated for $C_{38}H_{41}N_{10}O_5$: 717.3261, found: 717.3264.

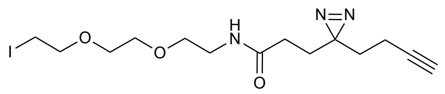

S7

3-(3-(but-3-yn-1-yl)-3*H*-diazirin-3-yl)-*N*-(2-(2-(2-iodoethoxy)ethoxy)ethyl)propanamide (S7): 3-[3-(but-3-yn-1-yl)-3H-diazirin-3-yl]propanoic acid **S8** (18 mg, 0.10 mmol) and *N*,*N*-diisopropylethylamine (19 μL, 0.15 mmol) were dissolved in dry CH_2Cl_2. HATU (46 mg, 0.12 mmol) was added and the mixture was stirred at room temperature for 1 h. 2-[2-(2-aminoethoxy)ethoxy]ethanol (23 μL, 0.15 mmol) was added and the reaction was stirred at room temperature overnight. The mixture was diluted with CH_2Cl_2 (10 mL). The solution was washed with 1 M HCl aq. (5 mL × 2) and brine (3 mL), dried over anhydrous $MgSO_4$, filtered and concentrated in vacuo. The crude product was used directly into next step. I_2 (40 mg, 0.15 mmol), triphenylphosphine (41 mg, 0.15 mmol), and imidazole (14 mg, 0.21 mmol) were dissolved in 3 mL dry CH_2Cl_2 and the mixture was stirred at room temperature for 10 min, and then the crude product from previous step was dissolved in 2 mL dry CH_2Cl_2 and added into the mixture. The reaction was stirred at room temperature for 3 h and subsequently quenched by sat. sodium thiosulfate solution (3 mL) and sat. $NaHCO_3$ solution (3 mL). The organic layer was separated out and the aqueous layer was extracted with CH_2Cl_2 (10 mL × 2). The combined organic layers were washed with brine, dried over anhydrous $MgSO_4$ and filtered. The solvents were removed in vacuo and the residue was purified by flash silica column chromatography (0% ~ 50% EtOAc in petroleum ether) to give the title compound **S7** as a colorless oil (17 mg, 39 μmol, 41% yield over two steps): ^{1}H NMR (400 MHz $CDCl_3$): δ 5.98 (br, 1H), 3.76 (t, *J* = 6.4 Hz, 2H), 3.68 – 3.62 (m, 4H), 3.57 (t,

J = 4.8 Hz, 2H), 3.45 (q, J = 5.2 Hz, 2H), 2.06 – 1.93 (m, 5H), 1.84 (t, J = 7.6 Hz, 2H), 1.65 (t, J = 7.6 Hz, 2H); ^{13}C NMR (100 MHz $CDCl_3$): δ 171.1, 82.7, 71.8, 70.2, 70.0, 69.7, 69.2, 39.3, 32.3, 30.4, 28.4, 27.8, 13.7, 2.9; HRMS (ESI-TOF): $[M+Na]^+$ calculated for $C_{14}H_{22}IN_3NaO_3$: 430.0604, found: 430.0601.

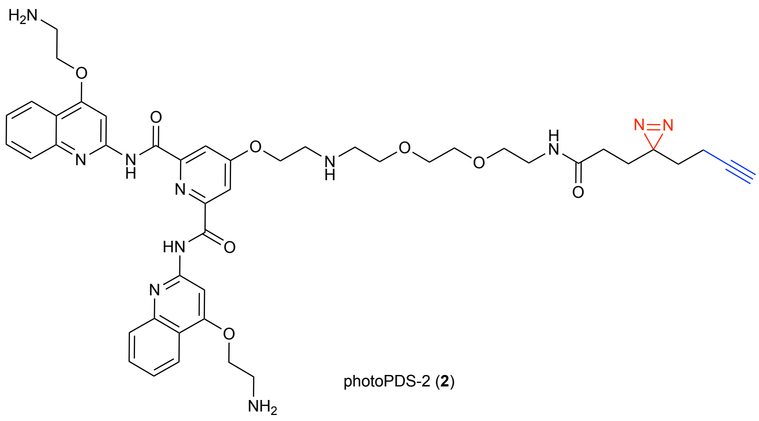

photoPDS-2 (**2**)

PhotoPDS-2 (2): S5 (47 mg, 59 µmol) was dissolved in 5 mL DMF and appropriate heating could be used if not completely dissolved. **S6** (34 mg, 0.083 mmol) and triethylamine (35 µL, 0.251 mmol) were added. The reaction mixture was stirred at 60 °C for 24 h. The solvent was removed in vacuo and the crude product was used directly into next step. The crude product was dissolved in 2 mL CH_2Cl_2 and 1 mL TFA was added. The mixture was stirred at room temperature for 30 min. The solvents were removed in vacuo and the residue was purified by HPLC (5% ~ 100% acetonitrile (with 0.1% TFA) in water (with 0.1% TFA) over 30 min). The most acetonitrile was removed in vacuo, and the rest of solvents were removed through freeze-drying to give the TFA salt of **2** as a white solid (21 mg, 24 µmol, 41% yield over two steps): ^{1}H NMR (500 MHz, Methanol-d_4) δ 8.51 (dd, J = 8.5 Hz, 1.5 Hz, 2H), 8.22 (s, 2H), 8.20 (s, 2H), 8.07 (dd, J = 9.0 Hz, 1.0 Hz, 2H), 7.95 (ddd, J = 8.5 Hz, 7.0 Hz, 1.5 Hz, 2H), 7.69 (ddd, J = 9.0 Hz, 7.0 Hz, 1.0 Hz, 2H), 4.77 (t, J = 4.5 Hz, 4H), 4.71 (t, J = 4.5 Hz, 2H), 3.85 (t, J = 4.5 Hz, 2H), 3.73 – 3.66 (m, 10H), 3.58 (t, J = 6.0 Hz, 2H), 3.42 (t, J = 5.5 Hz, 2H), 3.37 (t, J = 5.5 Hz, 2H), 2.25 (t, J = 2.5 Hz, 1H), 2.02 (t, J = 7.5 Hz, 2H), 1.98 (td, J = 7.5 Hz, 2.5 Hz, 2H), 1.72 (t, J = 8.0 Hz, 2H), 1.58 (t, J = 7.5 Hz, 1H); ^{13}C NMR (125 MHz, Methanol-d_4) δ 174.6, 169.1, 166.4, 164.1, 152.6, 151.4, 144.3, 133.9, 127.5, 125.0, 124.0, 120.3, 114.1, 95.4, 83.6, 71.5, 71.3, 70.6, 70.4, 67.4, 66.8, 65.8, 47.5, 40.3, 39.8, 33.3, 31.0, 29.8, 28.9, 13.8; HRMS (ESI-TOF): $[M+H]^+$ calculated for $C_{49}H_{55}N_8O_{11}$: 876.4157, found: 876.4138.

NMR spectra

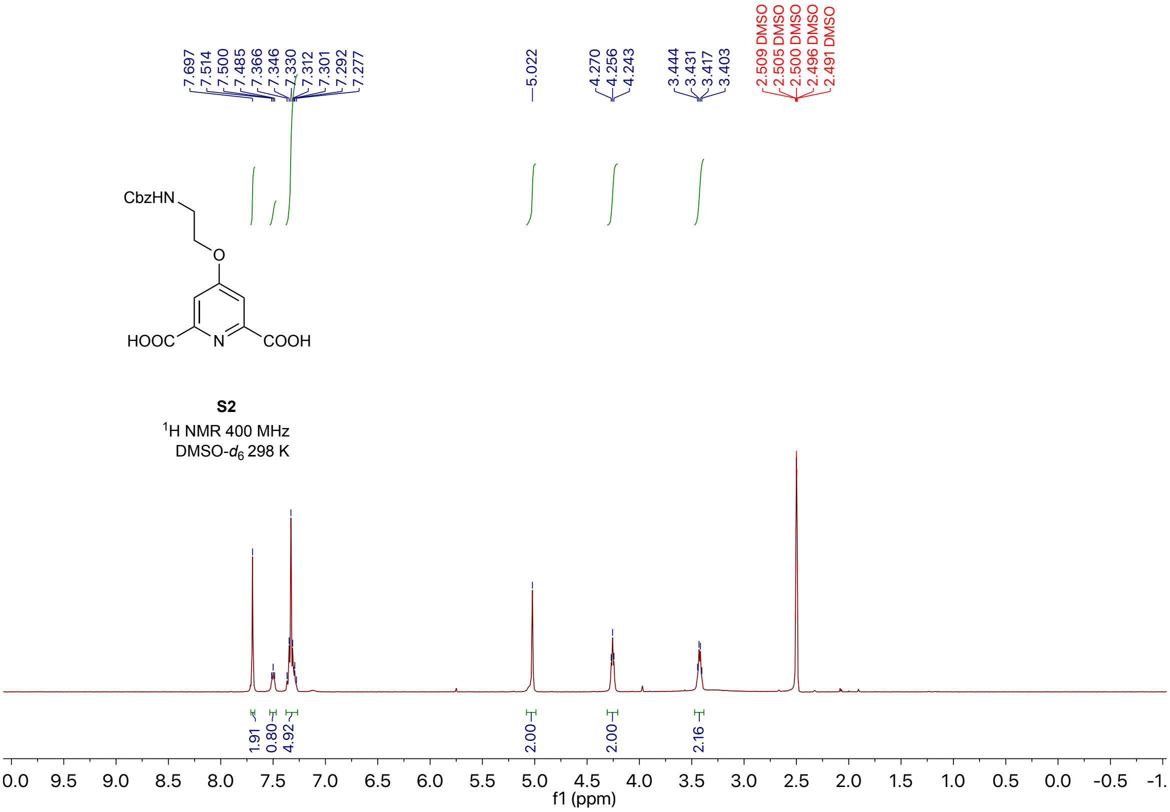

S2

^{1}H NMR 400 MHz

DMSO-d_6 298 K

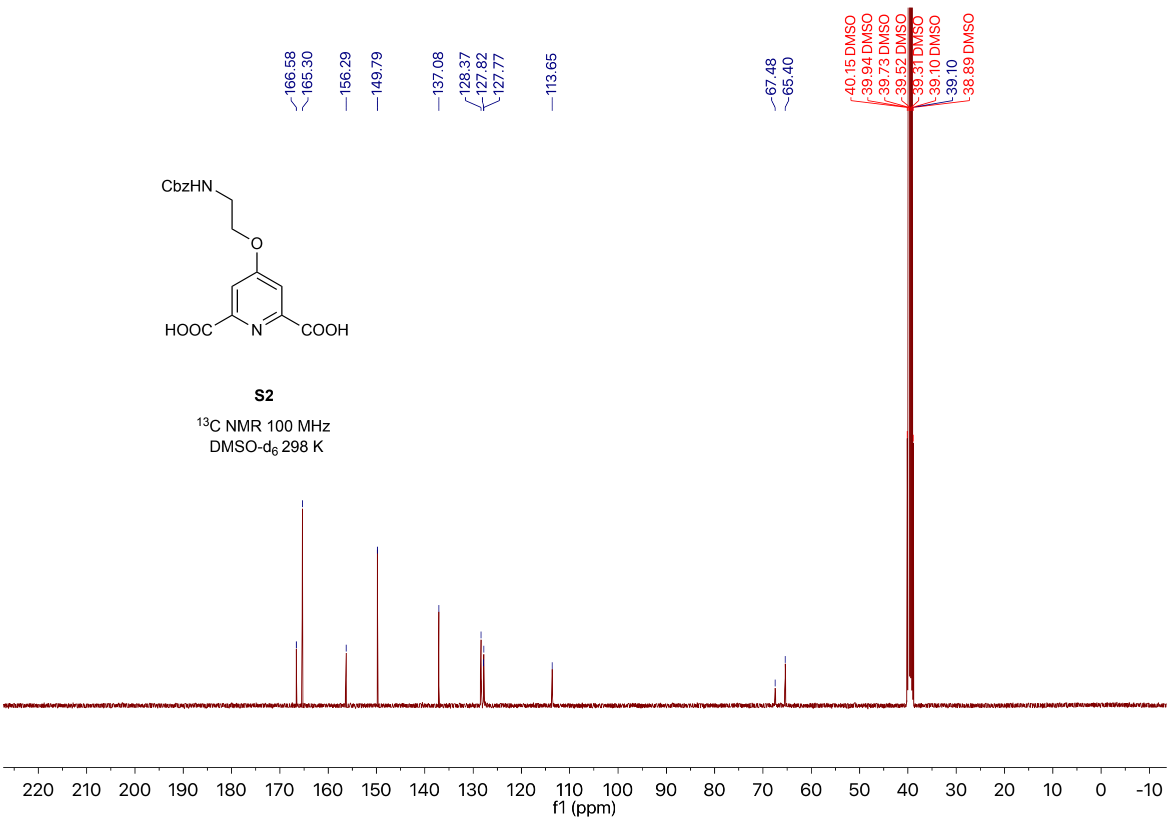

S2

^{13}C NMR 100 MHz

DMSO-d_6 298 K

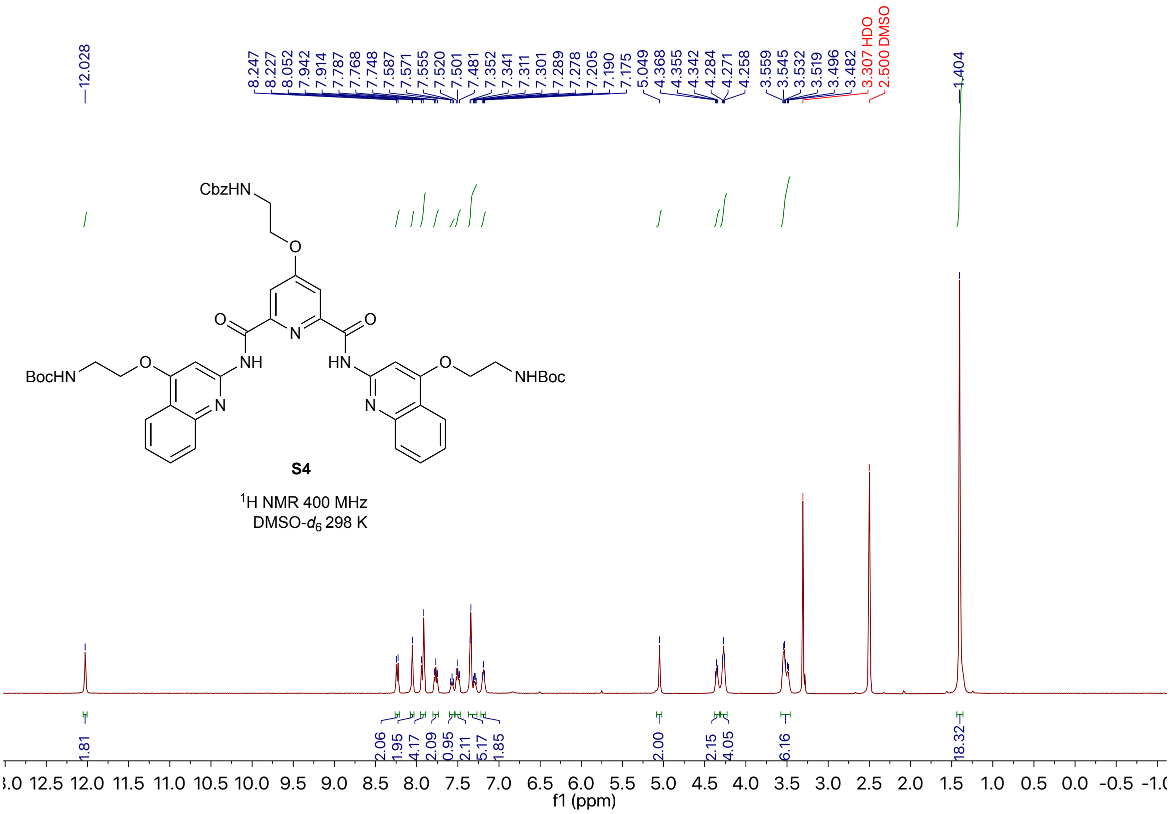
12.028
8.247 8.227 8.052 7.942 7.914 7.787 7.768 7.748 7.587 7.571 7.555 7.520 7.501 7.481 7.352 7.341 7.311 7.301 7.289 7.278 7.205 7.190 7.175
5.049 4.368 4.355 4.342 4.284 4.271 4.258 3.559 3.545 3.532 3.519 3.496 3.482
3.307 HDO
2.500 DMSO
1.404
CbzHN
O
O
N
O
BocHN
O
NH
HN
O
NHBoc
N
N
S4
^{1}H NMR 400 MHz
DMSO-d_6 298 K
1.81 2.06 1.95 4.17 2.09 0.95 2.11 5.17 1.85 2.00 2.15 4.05 6.16 18.32
f1 (ppm)

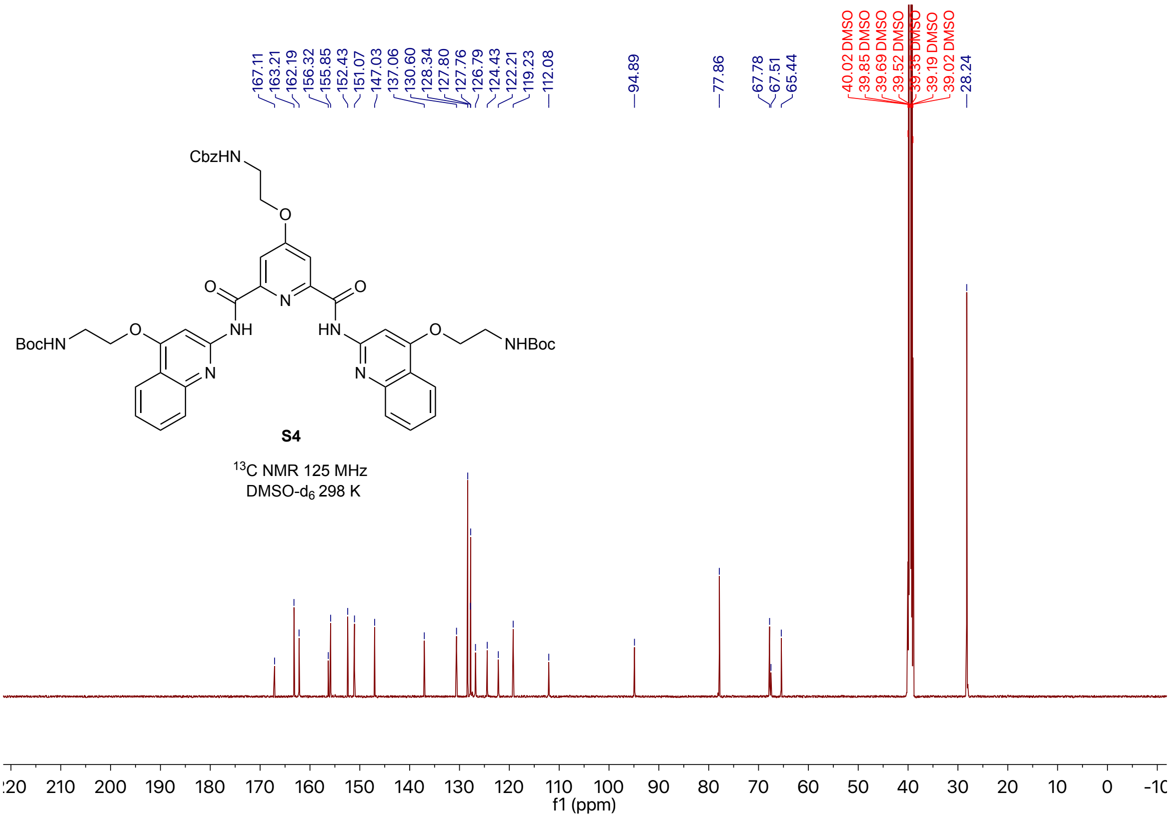
167.11 163.21 162.19 156.32 155.85 152.43 151.07 147.03 137.06 130.60 128.34 127.80 127.76 126.79 124.43 122.21 119.23 112.08
94.89
77.86
67.78 67.51 65.44
40.02 DMSO 39.85 DMSO 39.69 DMSO 39.52 DMSO 39.35 DMSO 39.19 DMSO 39.02 DMSO
28.24
CbzHN
O
O
N
O
BocHN
O
NH
HN
O
NHBoc
N
N
S4
^{13}C NMR 125 MHz
DMSO-d$_6$ 298 K
f1 (ppm)

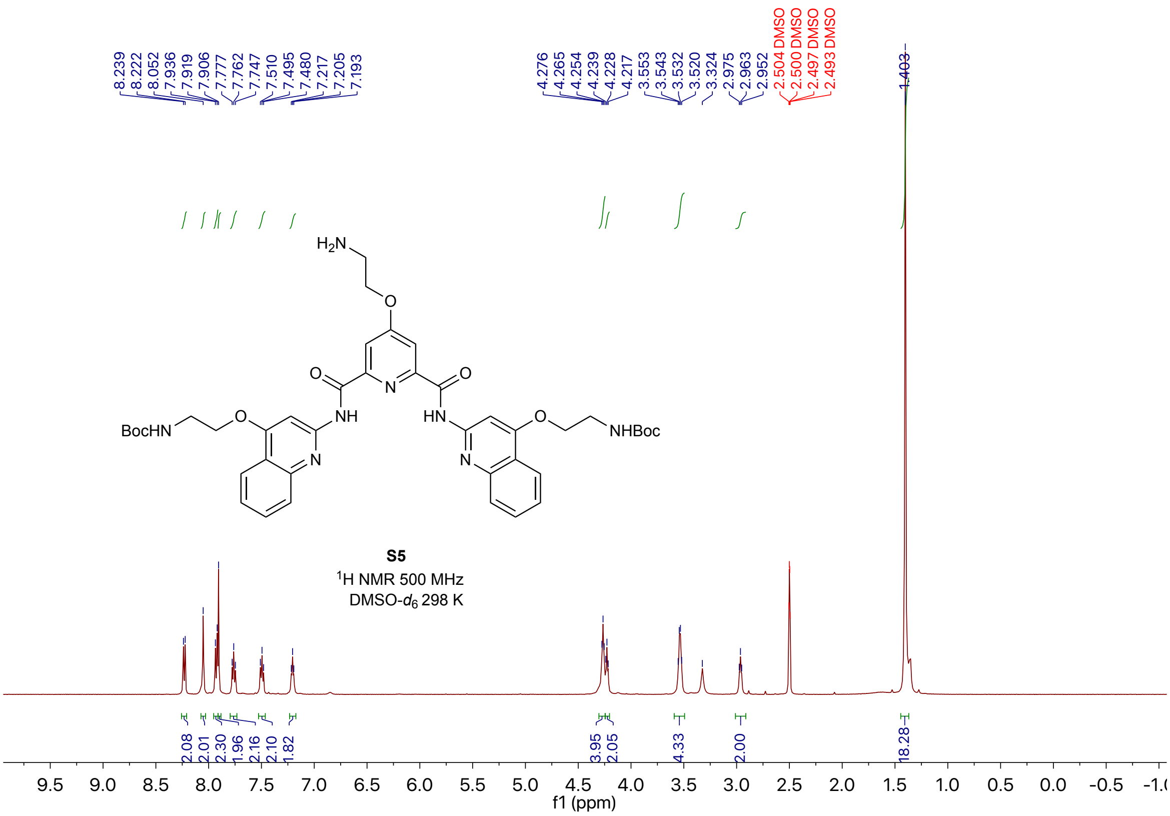
8.239
8.222
8.052
7.936
7.919
7.906
7.777
7.762
7.747
7.510
7.495
7.480
7.217
7.205
7.193
4.276
4.265
4.254
4.239
4.228
4.217
3.553
3.543
3.532
3.520
3.324
2.975
2.963
2.952
2.504 DMSO
2.500 DMSO
2.497 DMSO
2.493 DMSO
1.403
H2N
O
N
O
O
NH
HN
BocHN
O
O
NHBoc
N
N
S5
1H NMR 500 MHz
DMSO-d6 298 K
2.08
2.01
2.30
1.96
2.16
2.10
1.82
3.95
2.05
4.33
2.00
18.28
9.5 9.0 8.5 8.0 7.5 7.0 6.5 6.0 5.5 5.0 4.5 4.0 3.5 3.0 2.5 2.0 1.5 1.0 0.5 0.0 -0.5
f1 (ppm)

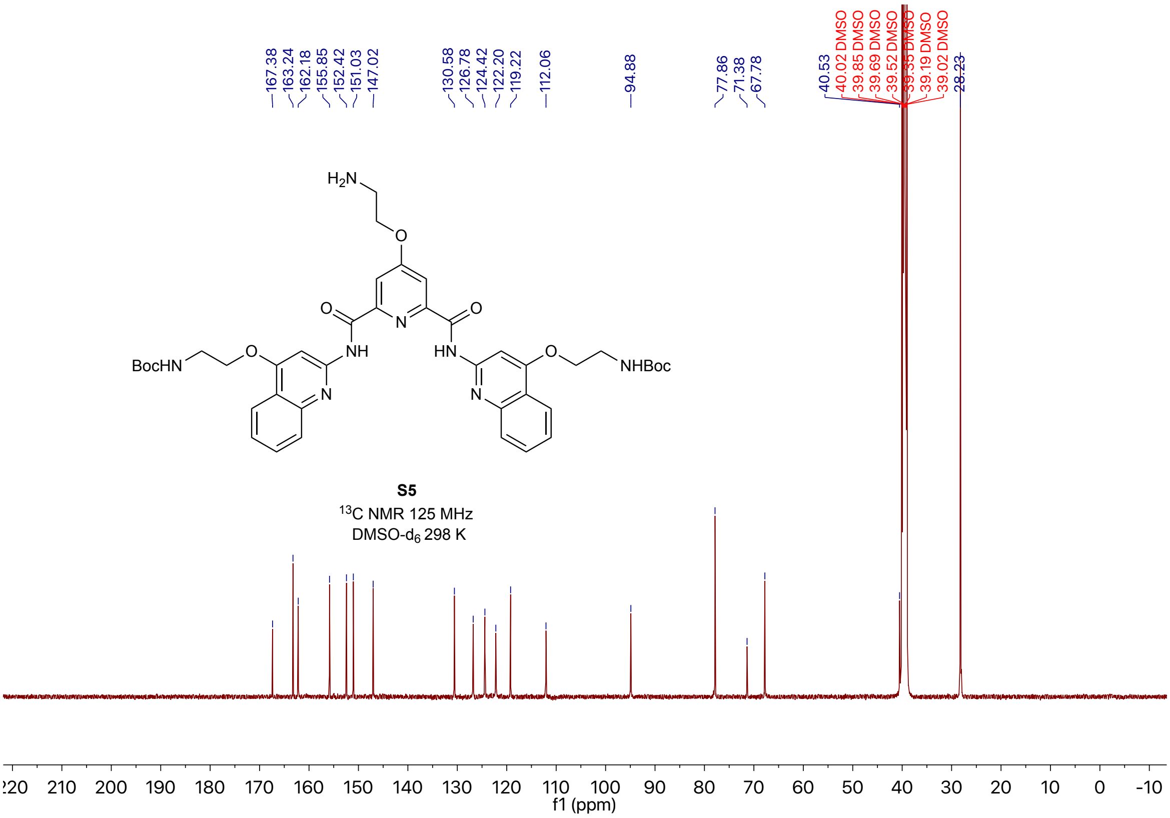
167.38
163.24
162.18
155.85
152.42
151.03
147.02
130.58
126.78
124.42
122.20
119.22
112.06
94.88
77.86
71.38
67.78
40.53
40.02 DMSO
39.85 DMSO
39.69 DMSO
39.52 DMSO
39.35 DMSO
39.19 DMSO
39.02 DMSO
28.23
H2N
O
N
O
O
NH
HN
BocHN
O
O
NHBoc
N
N
S5
13C NMR 125 MHz
DMSO-d6 298 K
210 200 190 180 170 160 150 140 130 120 110 100 90 80 70 60 50 40 30 20 10 0 -10
f1 (ppm)

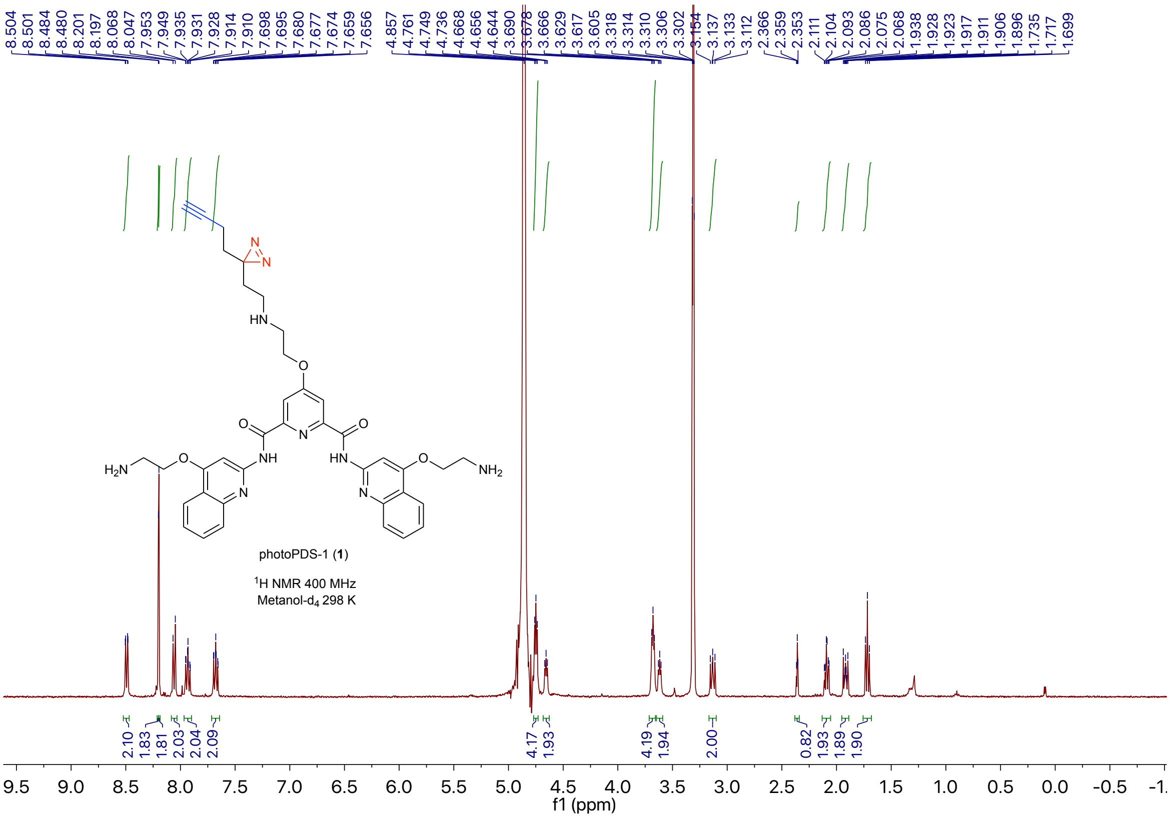
8.504 8.501 8.484 8.480 8.201 8.197 8.068 8.047 7.953 7.949 7.935 7.931 7.928 7.914 7.910 7.698 7.695 7.680 7.677 7.674 7.659 7.656 4.857 4.761 4.749 4.736 4.668 4.656 4.644 3.690 3.678 3.666 3.629 3.617 3.605 3.318 3.314 3.310 3.306 3.302 3.151 3.137 3.133 3.112 2.366 2.359 2.353 2.111 2.104 2.093 2.086 2.075 2.068 1.938 1.928 1.923 1.917 1.911 1.906 1.896 1.735 1.717 1.699
HN
O
N
O
O
NH
HN
H_2N
O
O
NH_2
N
N
photoPDS-1 (1)
^{1}H NMR 400 MHz
Metanol-d$_4$ 298 K
2.10 1.83 1.81 2.03 2.04 2.09 4.17 1.93 4.19 1.94 2.00 0.82 1.93 1.89 1.90
f1 (ppm)

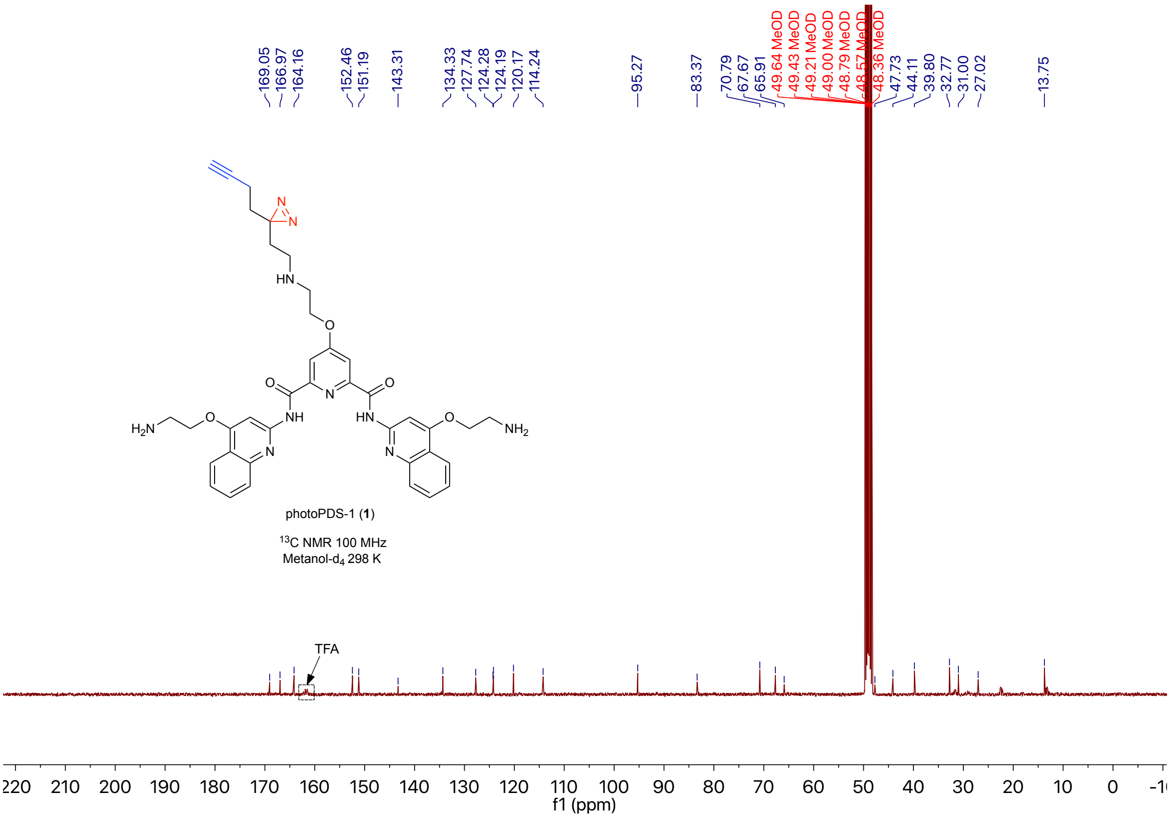
169.05 166.97 164.16 152.46 151.19 143.31 134.33 127.74 124.28 124.19 120.17 114.24 95.27 83.37 70.79 67.67 65.91 49.64 MeOD 49.43 MeOD 49.21 MeOD 49.00 MeOD 48.79 MeOD 48.57 MeOD 48.36 MeOD 47.73 44.11 39.80 32.77 31.00 27.02 13.75
HN
O
N
O
O
NH
HN
H_2N
O
O
NH_2
N
N
photoPDS-1 (1)
^{13}C NMR 100 MHz
Metanol-d$_4$ 298 K
TFA
f1 (ppm)

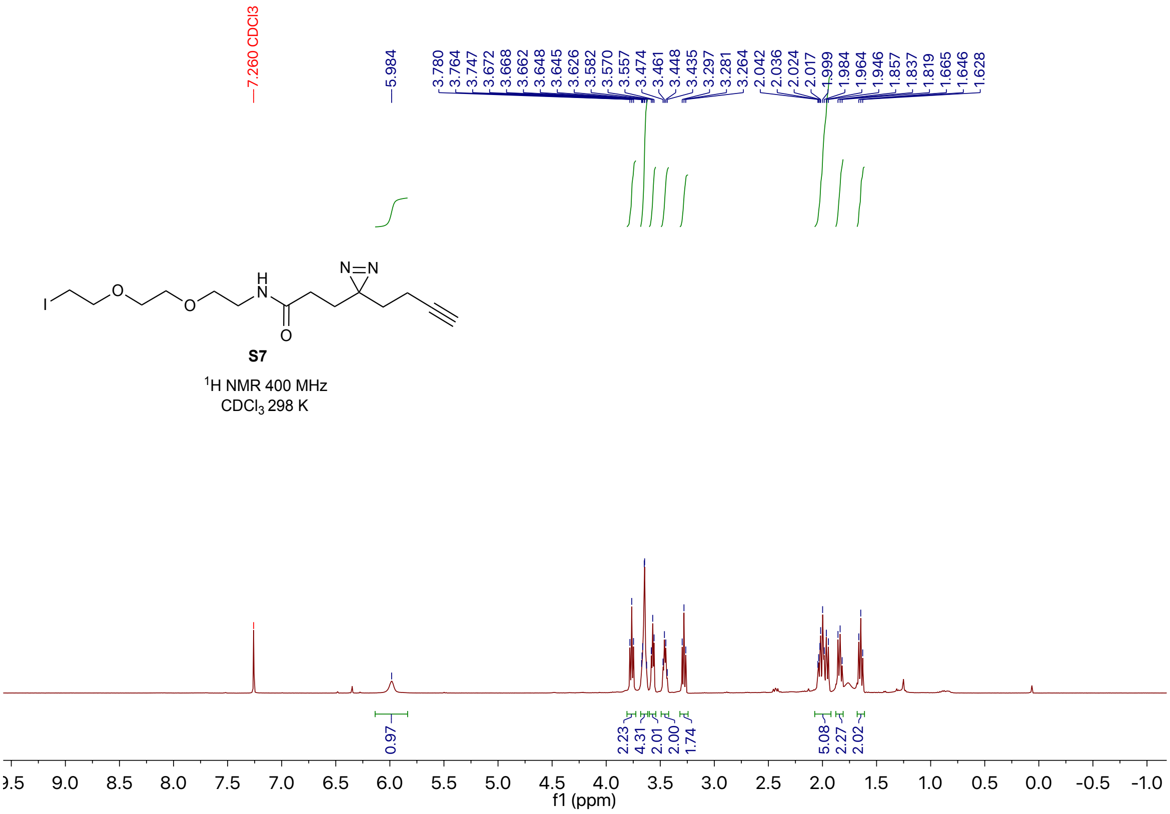
7.260 CDCl3
5.984
3.780
3.764
3.747
3.672
3.668
3.662
3.648
3.645
3.626
3.582
3.570
3.557
3.474
3.461
3.448
3.435
3.297
3.281
3.264
2.042
2.036
2.024
2.017
1.999
1.984
1.964
1.946
1.857
1.837
1.819
1.665
1.646
1.628
S7
^{1}H NMR 400 MHz
$CDCl_3$ 298 K
0.97
2.23
4.31
2.01
2.00
1.74
5.08
2.27
2.02
f1 (ppm)

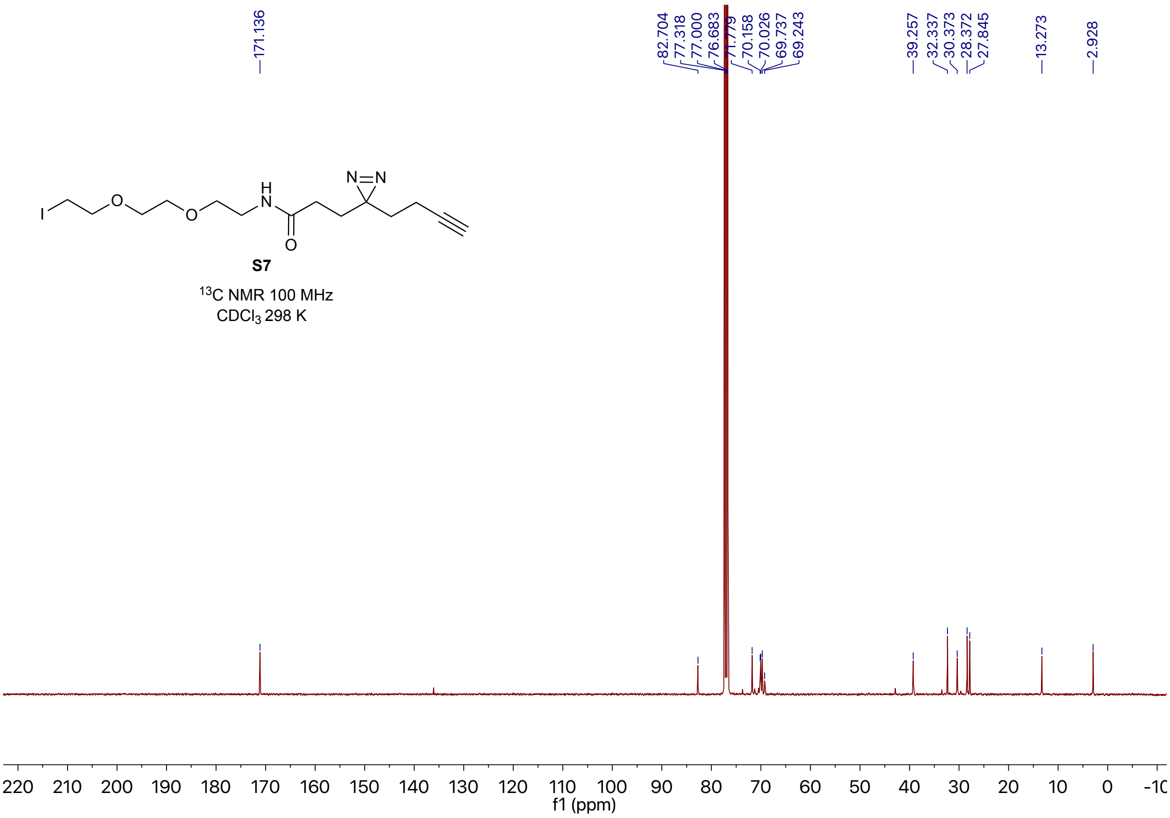
171.136
82.704
77.318
77.000
76.683
71.779
70.158
70.026
69.737
69.243
39.257
32.337
30.373
28.372
27.845
13.273
2.928
S7
^{13}C NMR 100 MHz
$CDCl_3$ 298 K
f1 (ppm)

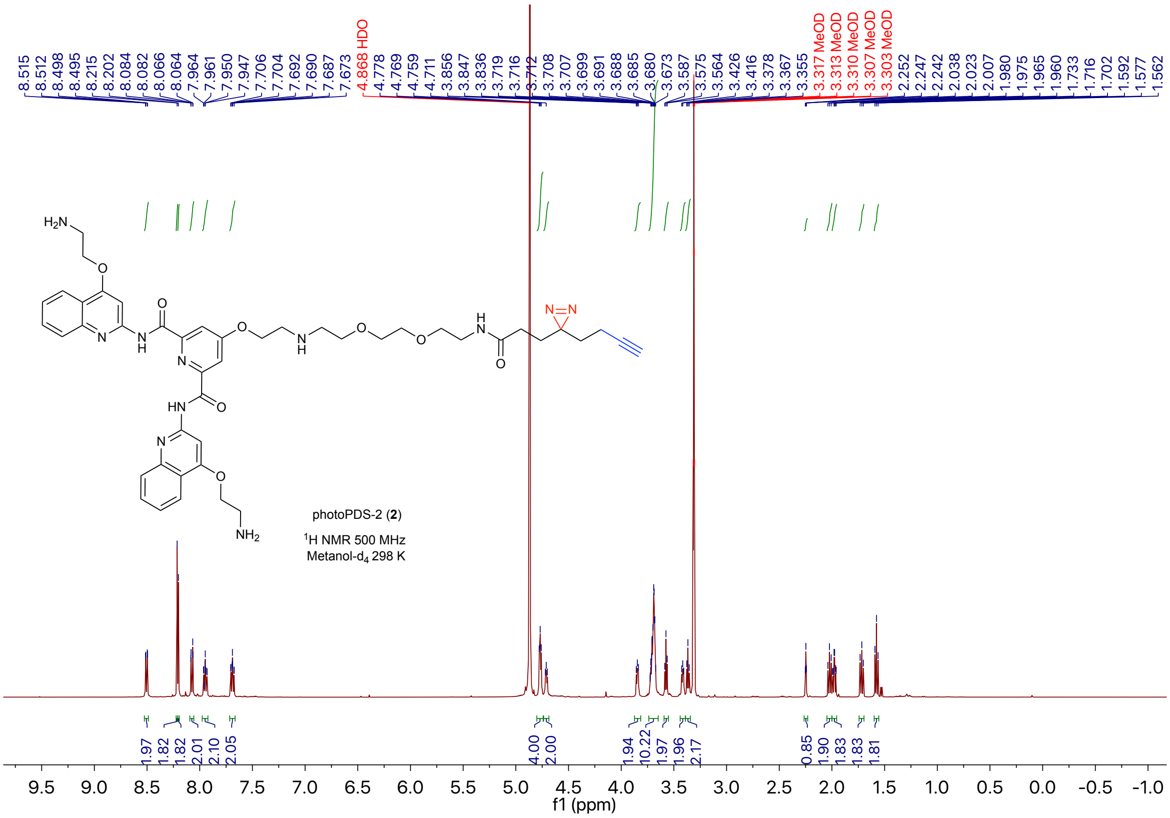
8.515 8.512 8.498 8.495 8.215 8.202 8.084 8.082 8.066 8.064 7.964 7.961 7.950 7.947 7.706 7.704 7.692 7.690 7.687 7.673
4.868 HDO
4.778 4.769 4.759 4.711 3.856 3.847 3.836 3.719 3.716 3.712 3.708 3.707 3.699 3.691 3.688 3.685 3.680 3.673 3.587 3.575 3.564 3.426 3.416 3.378 3.367 3.355
3.317 MeOD 3.313 MeOD 3.310 MeOD 3.307 MeOD 3.303 MeOD
2.252 2.247 2.242 2.038 2.023 2.007 1.980 1.975 1.965 1.960 1.733 1.716 1.702 1.592 1.577 1.562
H2N
O
N
N
H
O
O
N
H
O
O
H
N
O
N=N
HN
O
N
O
NH2
photoPDS-2 (2)
1H NMR 500 MHz
Metanol-d4 298 K
1.97 1.82 1.82 2.01 2.10 2.05 4.00 2.00 1.94 10.22 1.97 1.96 2.17 0.85 1.90 1.83 1.83 1.81
9.5 9.0 8.5 8.0 7.5 7.0 6.5 6.0 5.5 5.0 4.5 4.0 3.5 3.0 2.5 2.0 1.5 1.0 0.5 0.0 -0.5 -1.0
f1 (ppm)

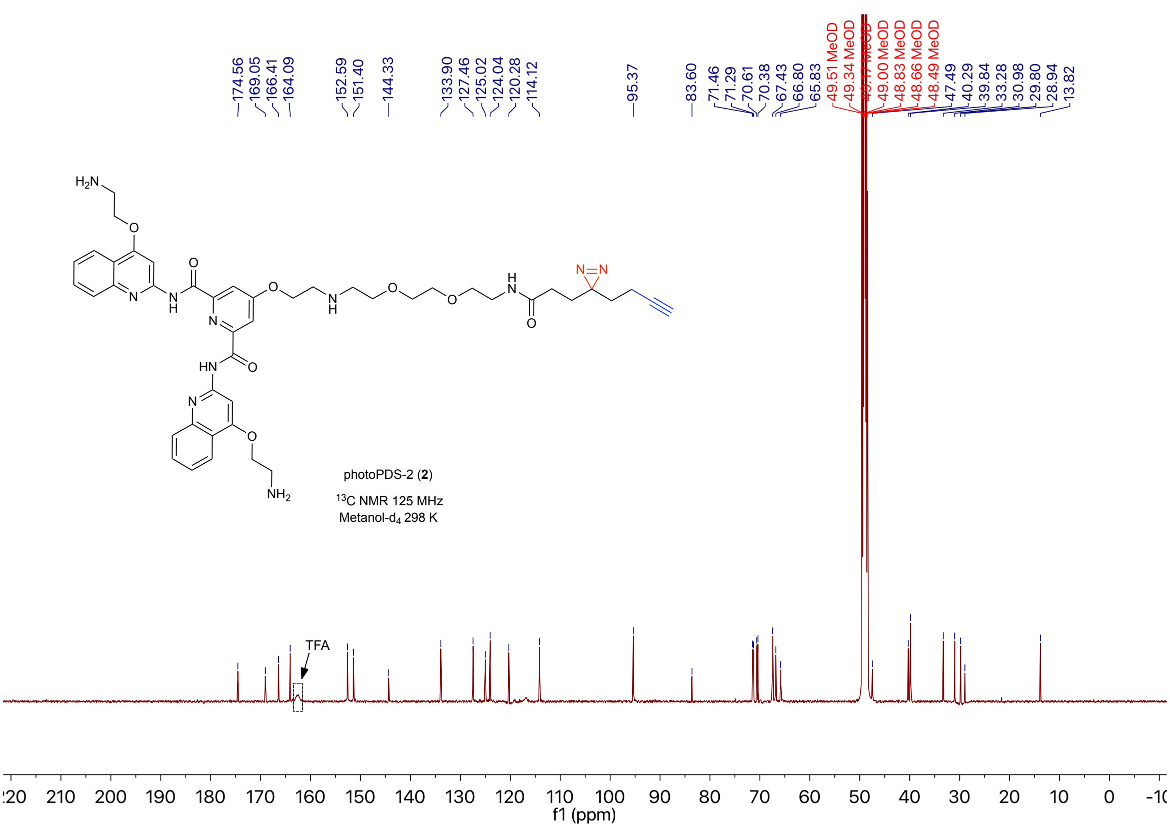
174.56 169.05 166.41 164.09 152.59 151.40 144.33 133.90 127.46 125.02 124.04 120.28 114.12 95.37 83.60 71.46 71.29 70.61 70.38 67.43 66.80 65.83
49.51 MeOD 49.34 MeOD 49.00 MeOD 48.83 MeOD 48.66 MeOD 48.49 MeOD
47.49 40.29 39.84 33.28 30.98 29.80 28.94 13.82
H2N
O
N
N
H
O
O
N
H
O
O
H
N
O
N=N
HN
O
N
O
NH2
photoPDS-2 (2)
13C NMR 125 MHz
Metanol-d4 298 K
TFA
220 210 200 190 180 170 160 150 140 130 120 110 100 90 80 70 60 50 40 30 20 10 0 -10
f1 (ppm)

References

1. Chauvin, A. S. et al. A versatile ditopic ligand system for sensitizing the luminescence of bimetallic lanthanide bio-imaging probes. *Chem. Eur. J.* **14**, 1726–1739 (2008).
2. Rodriguez, R. et al. A novel small molecule that alters shelterin integrity and triggers a DNA-damage response at telomeres. *J. Am. Chem. Soc.* **130**, 15758–15759 (2008).
3. Le, D. D., Di Antonio, M., Chan, L. K. M. & Balasubramanian, S. G-quadruplex ligands exhibit differential G-tetrad selectivity. *Chem. Commun.* **51**, 8048–8050 (2015).
4. Müller, S., Kumari, S., Rodriguez, R. & Balasubramanian, S. Small-molecule-mediated G-quadruplex isolation from human cells. *Nat. Chem.* **2**, 1095–1098 (2010).
5. Ambrus, A., Chen, D., Dai, J., Jones, R. A. & Yang, D. Solution structure of the biologically relevant G-quadruplex element in the human c-MYC promoter. Implications for G-quadruplex stabilization. *Biochemistry* **44**, 2048–2058 (2005).
6. Wei, D., Parkinson, G. N., Reszka, A. P. & Neidle, S. Crystal structure of a c-kit promoter quadruplex reveals the structural role of metal ions and water molecules in maintaining loop conformation. *Nucleic Acids Res.* **40**, 4691–4700 (2012).
7. Kuryavyi, V., Phan, A. T. & Patel, D. J. Solution structures of all parallel-stranded monomeric and dimeric G-quadruplex scaffolds of the human c-kit2 promoter. *Nucleic Acids Res.* **38**, 6757–6773 (2010).
8. Macaya, R. F., Schultze, P., Smith, F. W., Roe, J. A. & Feigon, J. Thrombin-binding DNA aptamer forms a unimolecular quadruplex structure in solution. *Proc. Natl. Acad. Sci. U. S. A.* **90**, 3745–3749 (1993).
9. Dai, J., Chen, D., Jones, R. A., Hurley, L. H. & Yang, D. NMR solution structure of the major G-quadruplex structure formed in the human BCL2 promoter region. *Nucleic Acids Res.* **34**, 5133–5144 (2006).
10. Biffi, G., Tannahill, D., McCafferty, J. & Balasubramanian, S. Quantitative visualization of DNA G-quadruplex structures in human cells. *Nat. Chem.* **5**, 182–186 (2013).
11. Zyner, K. G. et al. Genetic interactions of G-quadruplexes in humans. *Elife* **8**, e46793 (2019).
