# Supplementary material for: Chemical profiling of DNA G-quadruplex-interacting proteins in live cells
Source: Nat Chem. 2021 Jun 28;13(7):626–33. doi: 10.1038/s41557-021-00736-9 (PMC8245323; doi:10.1038/s41557-021-00736-9)
Supplement: Supplementary file 2 — Reporting Summary [file 41557_2021_736_MOESM24_ESM.pdf]

nature research

Corresponding author(s): Shankar Balasubramanian

Last updated by author(s): Apr 12, 2021

Reporting Summary

Nature Research wishes to improve the reproducibility of the work that we publish. This form provides structure for consistency and transparency in reporting. For further information on Nature Research policies, see our Editorial Policies and the Editorial Policy Checklist.

Statistics

For all statistical analyses, confirm that the following items are present in the figure legend, table legend, main text, or Methods section.

n/a	Confirmed	
☐	☒	The exact sample size (*n*) for each experimental group/condition, given as a discrete number and unit of measurement
☐	☒	A statement on whether measurements were taken from distinct samples or whether the same sample was measured repeatedly
☐	☒	The statistical test(s) used AND whether they are one- or two-sided<br>*Only common tests should be described solely by name; describe more complex techniques in the Methods section.*
☒	☐	A description of all covariates tested
☒	☐	A description of any assumptions or corrections, such as tests of normality and adjustment for multiple comparisons
☐	☒	A full description of the statistical parameters including central tendency (e.g. means) or other basic estimates (e.g. regression coefficient) AND variation (e.g. standard deviation) or associated estimates of uncertainty (e.g. confidence intervals)
☐	☒	For null hypothesis testing, the test statistic (e.g. *F*, *t*, *r*) with confidence intervals, effect sizes, degrees of freedom and *P* value noted<br>*Give P values as exact values whenever suitable.*
☒	☐	For Bayesian analysis, information on the choice of priors and Markov chain Monte Carlo settings
☒	☐	For hierarchical and complex designs, identification of the appropriate level for tests and full reporting of outcomes
☒	☐	Estimates of effect sizes (e.g. Cohen's *d*, Pearson's *r*), indicating how they were calculated

Our web collection on statistics for biologists contains articles on many of the points above.

Software and code

Policy information about availability of computer code

Data collection	Bruker 400 MHz Avance III HD Spectrometer, 500 MHz DCH Cryoprobe Spectrometer, Waters LCT Premier (ESI) spectrometer, Proteome Discoverer 2.2 (Thermo Scientific), Wes Protein Simple Western System with Compass for SW (4.0), Applied Photophysics Chirascan Plus Spectrometer, Bio-Rad CFX96 Touch Real-Time PCR Detection System, BMG PHERAstar Plus reader, BMG SPECTROstar nano microplate reader, Bio-Rad ChemiDoc MP system
Data analysis	NMR data was processed in MestReNova (version 12.0.1)<br>Gel analysis was performed with Image Lab (version 6.1.0)<br>Binding data for FRET melting assay, fluorescence quench binding assay, ELISA as well as cell viability data were processed with Prism 7 (GraphPad Software Inc.).<br>Capillary-based immunoassays on a Wes Protein Simple Western System were analysed using Compass for SW (4.0).<br>Bioinformatics data analyses and processing were performed using Bash, R and Python programming languages. The following tools were also used: cutadapt (v 1.16), BWA (v 0.7.15), Picard (v 2.14.0), MACS2 (v 2.1.1), bedtools57 (v 2.26.0), samtools (v 1.6), deepTools (v 3.1.2) and Intervene (0.6.4).<br>Scripts for proteomics and ChIP-seq analyses available in the github page dedicated to this study: https://github.com/sblab-bioinformatics/cmpp.

For manuscripts utilizing custom algorithms or software that are central to the research but not yet described in published literature, software must be made available to editors and reviewers. We strongly encourage code deposition in a community repository (e.g. GitHub). See the Nature Research guidelines for submitting code & software for further information.

Data

Policy information about availability of data

All manuscripts must include a data availability statement. This statement should provide the following information, where applicable:

- Accession codes, unique identifiers, or web links for publicly available datasets
- A list of figures that have associated raw data
- A description of any restrictions on data availability

The data reported in this study are available at the NCBI GEO repository under accession number GSE165124. Results from the proteomics analysis are included as Supplementary Data Set.

Field-specific reporting

Please select the one below that is the best fit for your research. If you are not sure, read the appropriate sections before making your selection.

☒ Life sciences ☐ Behavioural & social sciences ☐ Ecological, evolutionary & environmental sciences

For a reference copy of the document with all sections, see nature.com/documents/nr-reporting-summary-flat.pdf

Life sciences study design

All studies must disclose on these points even when the disclosure is negative.

Sample size	Initial in vitro validation of CMPP on BG4 were performed in three independent replicates.<br>Gel-based analyses of probe-labelled G4 interactomes were performed in three independent biological replicates.<br>MS-based proteomics analyses of probe-labelled G4 interactomes were performed in four independent biological replicates.<br>Initial studies screening of Protein-G4 interactions via pull-down affinity enrichment were performed two independent replicates.<br>ELISAs were performed in three independent replicates to ensure reproducibility.<br>ChIP-seq guidelines of the ENCODE consortia was followed. SMARCA4 ChIP-seq in K562 were performed in three biological replicates.<br>All experiments provided consistent and reproducible measurements.
Data exclusions	No data were excluded from analyses.
Replication	All experimental findings were reliably reproduced as described above.
Randomization	No randomization were applied.
Blinding	No animals or human participants were used in studies, and blinding was not used.

Reporting for specific materials, systems and methods

We require information from authors about some types of materials, experimental systems and methods used in many studies. Here, indicate whether each material, system or method listed is relevant to your study. If you are not sure if a list item applies to your research, read the appropriate section before selecting a response.

Materials & experimental systems

n/a	Involved in the study	
☐	☒	Antibodies
☐	☒	Eukaryotic cell lines
☒	☐	Palaeontology and archaeology
☒	☐	Animals and other organisms
☒	☐	Human research participants
☒	☐	Clinical data
☒	☐	Dual use research of concern

Methods

n/a	Involved in the study	
☐	☒	ChIP-seq
☒	☐	Flow cytometry
☒	☐	MRI-based neuroimaging

Antibodies

Antibodies used	Rabbit TTF2 polyclonal antibody (Proteintech, cat.#13722-1-AP), rabbit RBM22 polyclonal antibody (Proteintech, cat.#22103-1-AP), rabbit HMGB2 polyclonal antibody (Proteintech, cat.#14597-1-AP), rabbit monoclonal BRG1 antibody (abcam, cat.#ab110641), rabbit monoclonal UHRF1 antibody (abcam, cat.#ab194236), rabbit polyclonal DDX1 antibody (abcam, cat.#ab70252), rabbit polyclonal DDX24 antibody (abcam, cat.#ab70462), rabbit anti-GST HRP-conjugated antibody (abcam, cat.#ab3416), goat anti-FLAG HRP-conjugated antibody (abcam, cat.#ab1238), mouse anti-HIS HRP-conjugated antibody (BioLegend, cat.#652503), BG4.

Validation	Commercial antibodies had been validated by the manufacturers. Binding affinity and specificity of BG4 for G4 structures were confirmed by standard ELISA.

Eukaryotic cell lines

Policy information about cell lines

Cell line source(s)	Human embryonic kidney HEK293T cells (ATCC, CRL-3216) were provided by the CRUK Cambridge Institute Biorepository Core Facility. Human chronic myelogenous leukemia K562 cells (CCL-243) were purchased from ATCC.
Authentication	Short tandem repeat (STR) profiling was used to distinguish between individual human cell lines and rule out intra-species contamination performed by the CRUK Cambridge Institute Biorepository Core Facility.
Mycoplasma contamination	Cells were tested mycoplasma-free based on RNA-capture ELISA performed by the CRUK Cambridge Institute Biorepository Core Facility.
Commonly misidentified lines (See ICLAC register)	No commonly misidentified cell lines were used.

ChIP-seq

Data deposition

☒ Confirm that both raw and final processed data have been deposited in a public database such as GEO.

☒ Confirm that you have deposited or provided access to graph files (e.g. BED files) for the called peaks.

Data access links<br>*May remain private before publication.*	The data reported in this paper are available at the NCBI GEO repository under accession number GSE165124. The BG4 ChIP-seq data were generated in a previous study and are available under accession number GSE107690.
Files in database submission	K562_Rep1_Input_SLX-19726.NEBi708_NEBi508.HLVFVBGXG.s_1.r_1.fq.gz<br>K562_Rep1_SMARCA4_SLX-19726.NEBi709_NEBi502.HLVFVBGXG.s_1.r_1.fq.gz<br>K562_Rep2_Input_SLX-19727.NEBi701_NEBi501.HNL5GBGXG.s_1.r_1.fq.gz<br>K562_Rep2_SMARCA4_SLX-19727.NEBi707_NEBi507.HNL5GBGXG.s_1.r_1.fq.gz<br>K562_Rep3_Input_SLX-19727.NEBi702_NEBi502.HNL5GBGXG.s_1.r_1.fq.gz<br>K562_Rep3_SMARCA4_SLX-19727.NEBi708_NEBi508.HNL5GBGXG.s_1.r_1.fq.gz<br>K562_SMARCA4_rep1-3_mult.2of3.bed
Genome browser session (e.g. UCSC)	*Provide a link to an anonymized genome browser session for "Initial submission" and "Revised version" documents only, to enable peer review. Write "no longer applicable" for "Final submission" documents.*

Methodology

Replicates	Shared peaks for SMARCA4 ChIP-seq replicates are shown in Supplementary Fig. 5a.
Sequencing depth	ChIP-seq was performed in single-end mode with 75-bp read length. In accordance with ENCODE guidelines for TF and narrow-peak histone ChIP-seq experiments, we aimed for >30 Mio usable fragments per library.<br>K562_Rep1_Input_SLX-19726.NEBi708_NEBi508.bam 42,432,209<br>K562_Rep1_SMARCA4_SLX-19726.NEBi709_NEBi502.bam 46,790,214<br>K562_Rep2_Input_SLX-19727.NEBi701_NEBi501.bam 31,733,009<br>K562_Rep2_SMARCA4_SLX-19727.NEBi707_NEBi507.bam 49,046,604<br>K562_Rep3_Input_SLX-19727.NEBi702_NEBi502.bam 80,095,823<br>K562_Rep3_SMARCA4_SLX-19727.NEBi708_NEBi508.bam 41,693,339
Antibodies	Per replicate we employed 5 µg of ChIP-grade antibody against SMARCA4 (abcam, ab110641)
Peak calling parameters	macs2 callpeak --name ../macs2_output/${f%%.nodup.bam}.nodup.q005.all -t $f -c Input.nodup.bam --format=BAM --gsize 'hs' --bw=300 --qvalue 0.05
Data quality	(see also Supplementary Fig. 5a)<br>Individual peak numbers for qvalue <0.05:<br>21,487 Rep1_SMARCA4<br>27,356 Rep2_SMARCA4<br>40,707 Rep3_SMARCA4
Software	The following tools were used for ChIP-seq analysis: cutadapt (v 1.16), BWA (v 0.7.15), Picard (v 2.14.0), MACS2 (v 2.1.1), bedtools (v 2.26.0), samtools (v 1.6) and deepTools (v 3.1.2).
