# Supplementary figures and images for: Chemical profiling of DNA G-quadruplex-interacting proteins in live cells
Source: Nat Chem. 2021 Jun 28;13(7):626–33. doi: 10.1038/s41557-021-00736-9 (PMC8245323; doi:10.1038/s41557-021-00736-9)

Fig. 2e, left panel

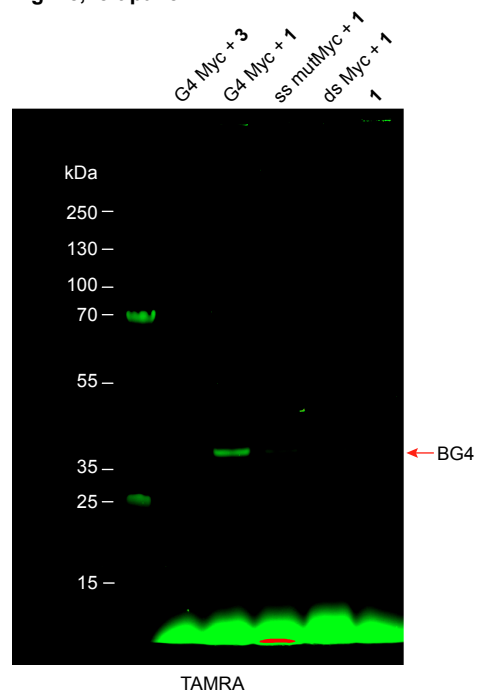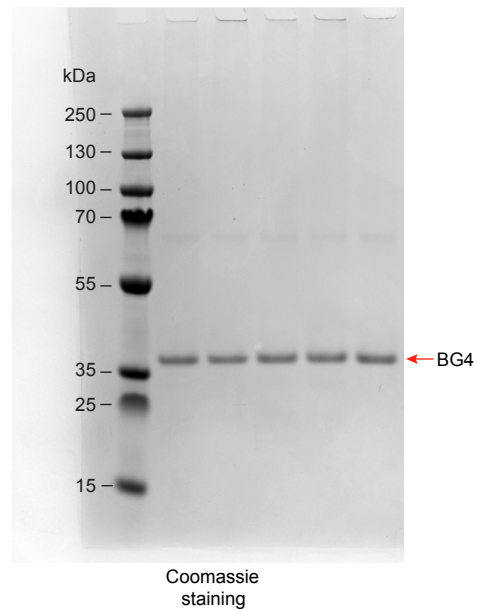

Fig. 2e, right panel

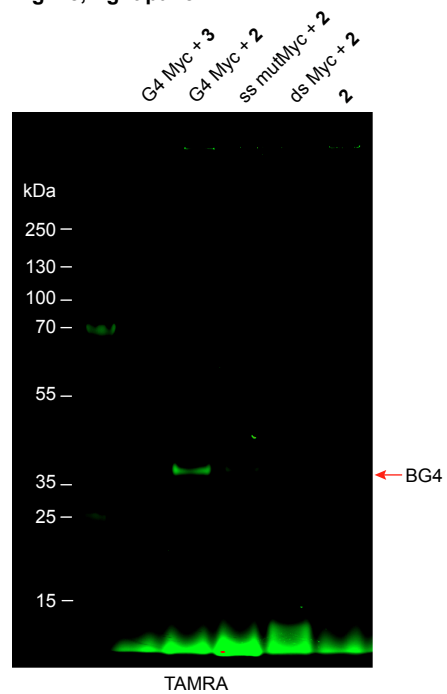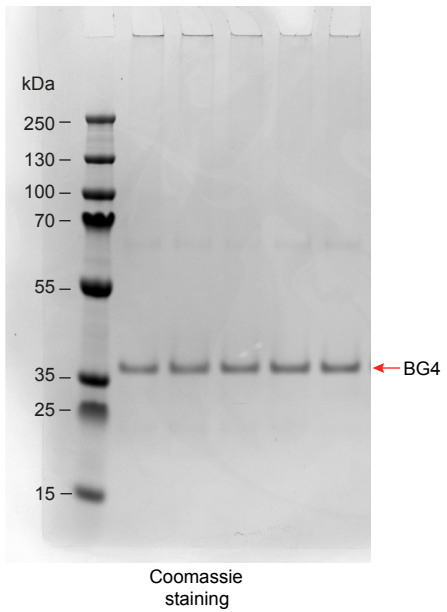

Supplement: Source Data Fig. 2 — Uncropped gels and statistical source data. [file 41557_2021_736_MOESM26_ESM.zip › Source Data Fig. 2/41557_2021_736_MOESM27_ESM.pdf]

**Fig. 3b**

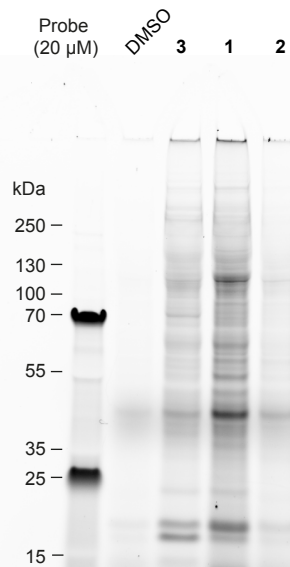

TAMRA

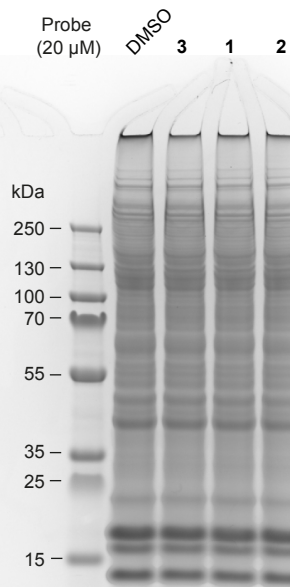

Coomassie staining

Supplement: Source Data Fig. 3 — Uncropped gels and statistical source data. [file 41557_2021_736_MOESM27_ESM.zip › Source Data Fig. 3/41557_2021_736_MOESM28_ESM.pdf]

**Fig. 4a**

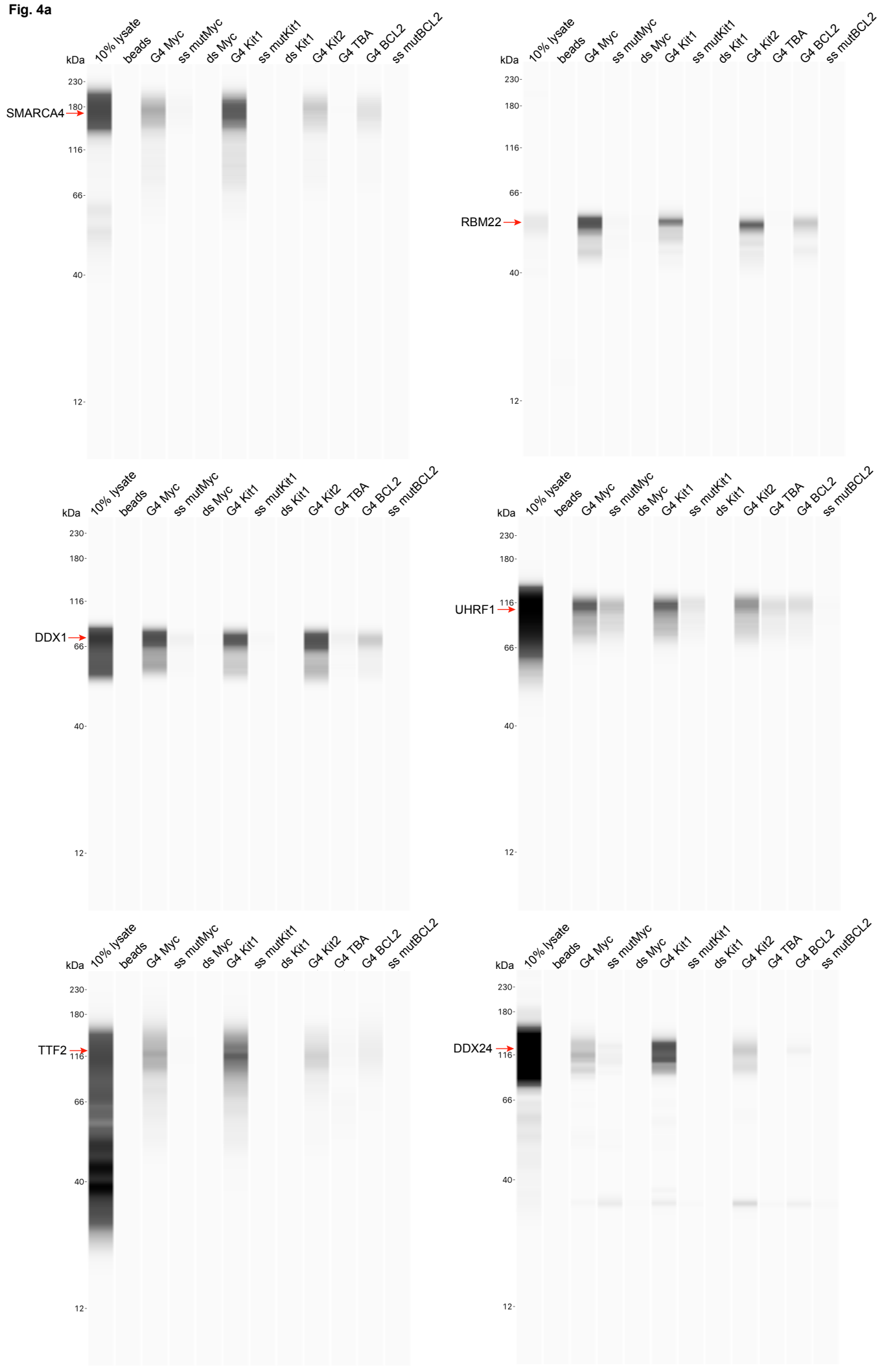

Supplement: Source Data Fig. 4 — Uncropped Western Blots and statistical source data. [file 41557_2021_736_MOESM28_ESM.zip › Source-Data_Fig.4_Uncropped-Western-Blots.pdf]

Extended Data Fig. 1d, left panel

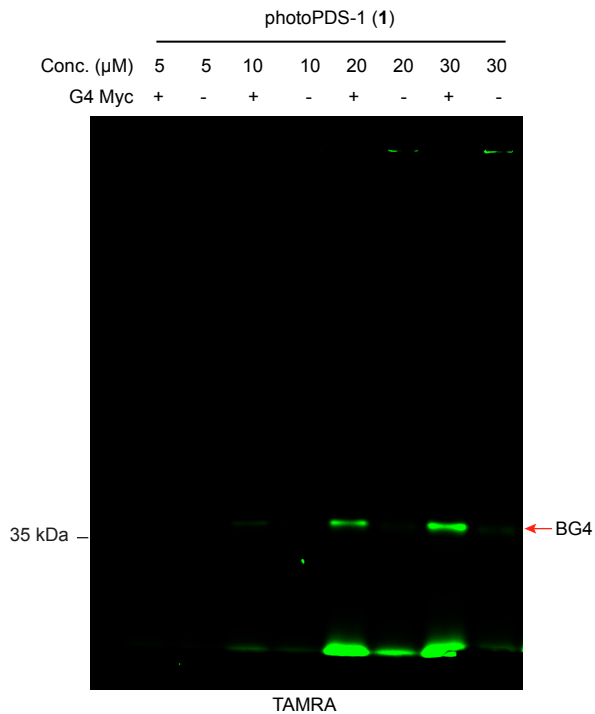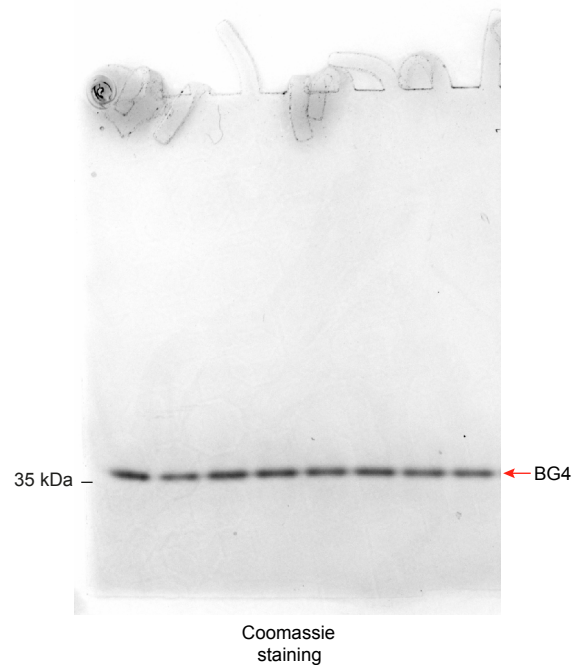

Extended Data Fig. 1d, right panel

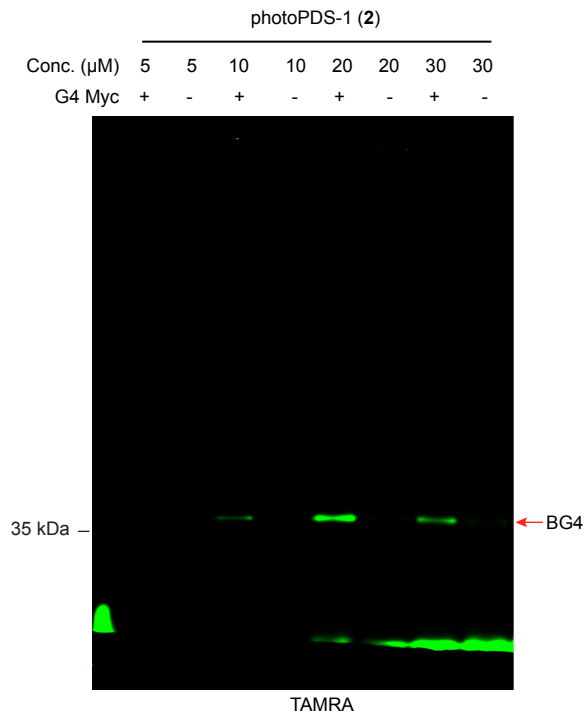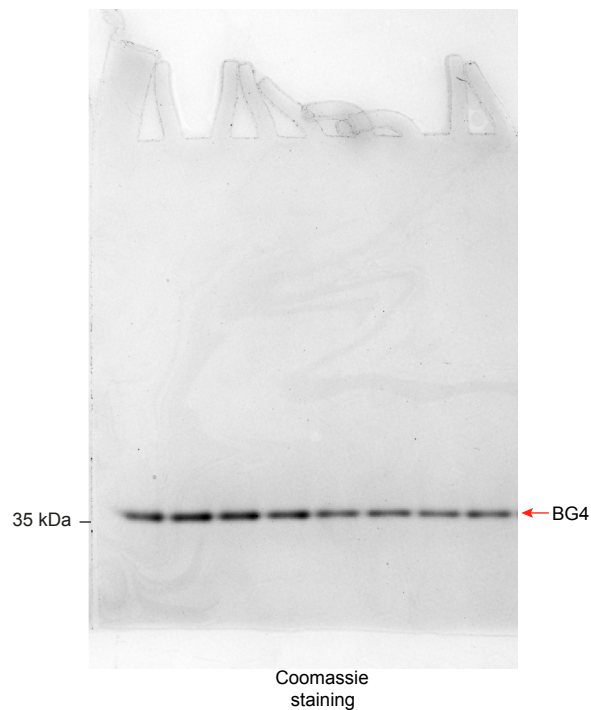

Supplement: Source Data Extended Data Fig. 1 — Uncropped gels and statistical source data. [file 41557_2021_736_MOESM30_ESM.zip › Source-Data_ED_Fig.1_Uncropped-gels.pdf]

Extended Data Fig. 2a

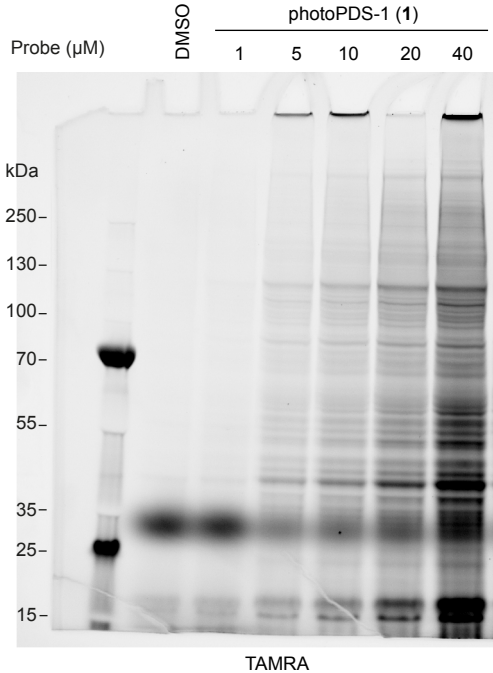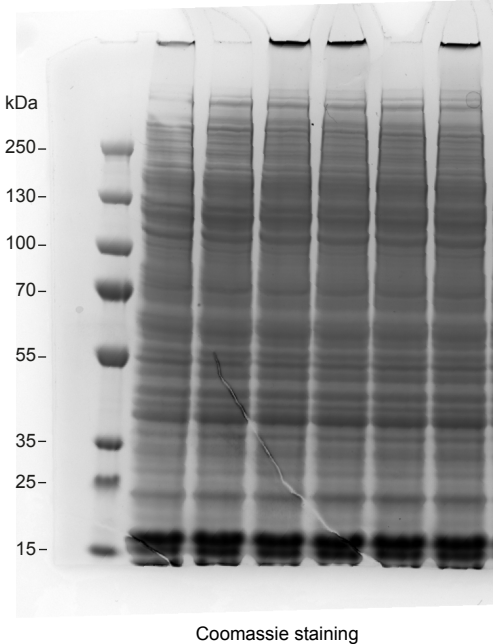

Extended Data Fig. 2b

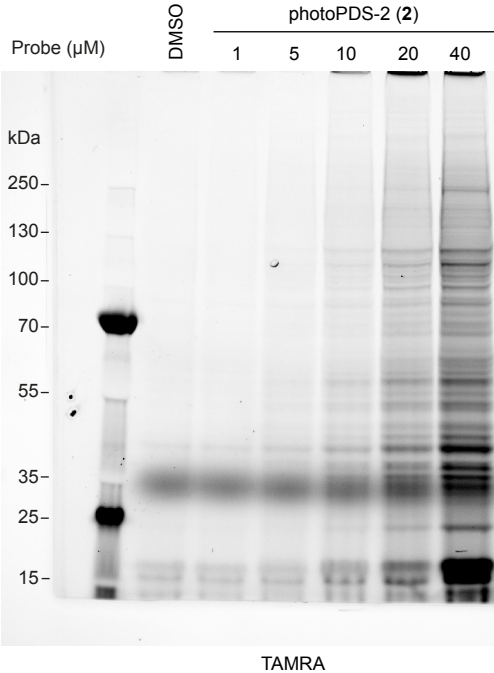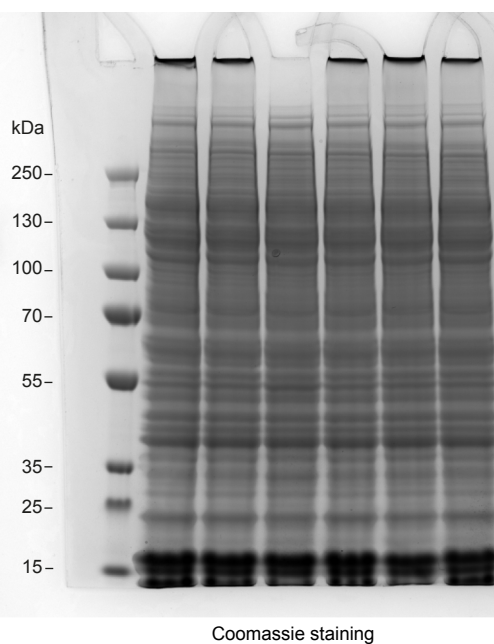

Supplement: Source Data Extended Data Fig. 2 — Uncropped gels and statistical source data. [file 41557_2021_736_MOESM31_ESM.zip › Source-Data_ED_Fig.2_Uncropped-gels.pdf]

Extended Data Fig. 4a

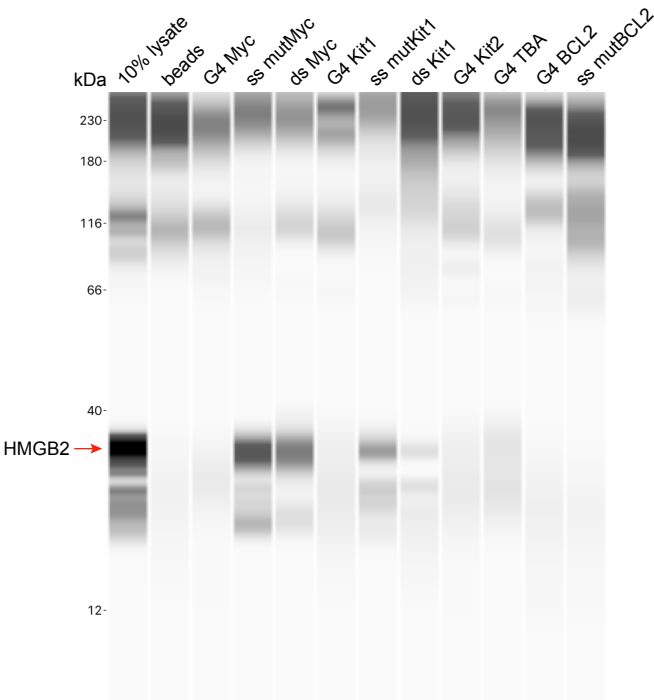

Supplement: Source Data Extended Data Fig. 4 — Uncropped Western Blots and statistical source data. [file 41557_2021_736_MOESM33_ESM.zip › Source-Data_ED_Fig.4_Uncropped-Western-Blots.pdf]
